# Author Correction: Gene isoforms as expression-based biomarkers predictive of drug response in vitro

**DOI:** 10.1038/s41467-017-02136-5

**Published:** 2018-01-09

**Authors:** Zhaleh Safikhani, Petr Smirnov, Kelsie L. Thu, Jennifer Silvester, Nehme El-Hachem, Rene Quevedo, Mathieu Lupien, Tak W. Mak, David Cescon, Benjamin Haibe-Kains

**Affiliations:** 1 Princess Margaret Cancer Centre, University Health Network, 101 College Street, Toronto, ON M5G1L7 Canada; 2 Department of Medical Biophysics, University of Toronto, 101 College Street, Toronto, ON M5G1L7 Canada; 3 Institut de recherches cliniques de Montréal, 110 Pine Avenue West, Montreal, QC H2W 1R7 Canada; 4 Campbell Family Institute for Breast Cancer Research, 620 University Avenue, Toronto, ON M5G2C1 Canada; 5 Division of Medical Oncology and Hematology, Department of Medicine, University of Toronto, 27 King’s College Circle, Toronto, ON M5S 1A1 Canada; 6 Department of Computer Science, University of Toronto, 10 King’s College Road, Toronto, ON M5S 3G4 Canada; 7 Ontario Institute of Cancer Research, 661 University Ave, Suite 510, Toronto, ON M5G 0A3 Canada

Correction to: *Nature Communications* 10.1038/s41467-017-01153-8, published online 24 October 2017

In the original version of this Article, financial support was not fully acknowledged. The PDF and HTML versions of the Article have now been corrected to include the following:

The research was supported by a Stand Up To Cancer Canada—Canadian Cancer Society Breast Cancer Dream Team Research Funding, with supplemental support of the Ontario Institute for Cancer Research through funding provided by the Government of Ontario (Funding Award Number: SU2C-AACR-DT-18-15). Stand Up To Cancer Canada is a program of the Entertainment Industry Foundation Canada. Research funding is administered by the American Association for Cancer Research International—Canada, the Scientific Partner of SU2C Canada.

