# Supplementary material for: Gene isoforms as expression-based biomarkers predictive of drug response in vitro
Source: Nat Commun. 2017 Oct 24;8:1126. doi: 10.1038/s41467-017-01153-8 (PMC5655668; doi:10.1038/s41467-017-01153-8)
Supplement: Supplementary file 1 — Supplementary Information [file 41467_2017_1153_MOESM1_ESM.pdf]

Supplementary Methods

Cell line identity using SNP fingerprinting

To assess the identity of cell lines from GDSC and CCLE, data of low quality was first excluded from our comparative analysis as no conclusion can be drawn regarding the identity of the corresponding cell lines. Of the 973 CEL files from GDSC, only 66 fell below the 0.4 threshold (6.88%) for contrast QC scores, indicating issues in resolving base calls. Additionally, five of the 1,190 CEL files from CCLE had an absolute difference between contrast QC scores for Nsp and Sty fragments greater than 2, thus indicating some issues with the efficacy of one enzyme set during sample preparation. CEL files with contrast QC scores indicative of some sort of issue with the assay that would affect the genotype call rate or birdseed accuracy were removed and genotype calling was conducted on the remaining CEL files using Birdseed version 2. The resulting files were then filtered to keep only the 1006 SNP fingerprints that originated from CEL files that had a common cell line annotation between GDSC and CCLE (503 CEL files from each). Finally, pairwise concordances of all SNP fingerprints were generated according to the method outlined by Hong et al. [1]. Genentech did use SNP fingerprinting to check the identity of their cell line panel against external databases (668 cell lines, referred to as gCSI). We sought to use the same approach than the one we used for GDSC and CCLE by using the published SNP calls generated using the Illumina HumanOmni 2.5M quad v1 platform [2]. Focusing on the set of 2,295,239 high-quality SNPs, we were able to match the gCSI cell lines with those of GDSC and CCLE; however the lack of metadata for the SNP array data prevented us from confirming their identity independently.

Quality controls of drug dose-response curves

Assuming D_i and $\Delta(i, i+1)$ to be the viability of a cell in presence of ith drug concentration tested in a drug sensitivity experiment, and the difference of the cell viability measures in the presence of the $(i+1)^{st}$ and i^{th} drug concentrations, respectively. The trend of a drug-response curve is expected to be non-increasing. In order to have a non-noisy curve, one can assume that the $\Delta(i, i+1)$ must be less than a small positive threshold (ϵ) in a large fraction of the cases(ρ) (1). Since noise is unavoidable in the cell culturing and drug sensitivity experiments, it is expected to see some fluctuations in viability values. As a result, considering very small values for ϵ yields to identifying many non-noisy curves as noisy incorrectly. Clearly choosing the best value for ϵ and ρ is a trade-off between sensitivity and specificity. However, applying only equation (1) on the drug response curve will not filter all the noisy cases out. For instance, assume a dose response curve with monotonically increasing viability from 99% to 100% over ρ percent of successive drug concentrations; but viability falls monotonically to 20% over the last concentrations. Equation (1) does not detect this curve as noisy. Therefore, we have to add other constraints to filter out these types of noisy curves. These constraints require the sum of the $\Delta(i, i+1 \forall i$ to be less than ϵ (2) and the sum of the $\Delta(i,j) \forall i, j$ to be less than 2ϵ (3).

$$\Delta(i,j) = D_j - D_i$$

$$\frac{|\{i|\Delta(i,i+1) < \epsilon\}|}{|\{D_i\}| - 1} > \rho(1)$$

$$\sum_{i} \Delta(i, i+1) < \epsilon (2)$$

$$\sum_{j-i>=2} \Delta(i, j) < 2\epsilon (3)$$

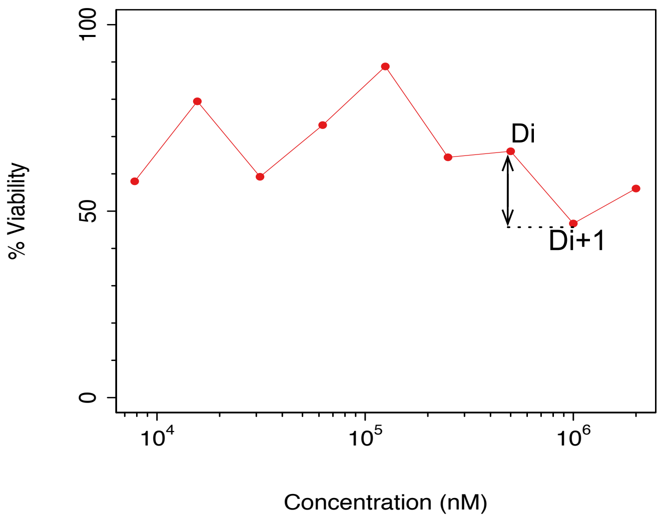

Drug dose-response curve fitting

All dose-response curves were fitted to the equation

$$y = E + 1 - E_1 + (\frac{x}{EC_{50}})^{HS}$$

where $y = 0$ denotes death of all infected cells, $y = y(0) = 1$ denotes no effect of the drug dose, EC_{50} is the concentration at which viability is reduced to half of the viability observed in the presence of an arbitrarily large concentration of drug, and HS is a parameter describing the cooperativity of binding. $HS < 1$ denotes negative binding cooperativity, $HS = 1$ denotes noncooperative binding, and $HS > 1$ denotes positive binding cooperativity. The parameters of the curves were fitted using the least squares optimization framework.

Concordance of CCLE RNA-seq and microarray profiles

To confirm validity of the CCLE RNA-seq data, we compared the expression profiles of identical and different cell lines from CCLE RNA-seq with the already published microarray profile of the cell lines (Supplementary Figure 4). Despite platform differences between RNA-seq and microarray data, identical cell lines are significantly more correlated than different cell lines, thereby confirming the validity of the CCLE RNA-seq data.

Recompute AUC values

Previous analyses across several pharmacogenomic studies [30,32] showed that the use of heterogeneous experimental protocols, including computational methods for estimating drug sensitivity, yields poor consistency across these studies. To mitigate these issues, we decided to apply a homogeneous drug sensitivity calculation approach for both the datasets. We have therefore downloaded drug responses data form for CCLE. These drug responses are indicating the cell viability percentage which have been calculated by normalizing the number of cells in compound-treated based on the median level of untreated wells. We also retrieved raw drug responses data for GDSC. We applied a CCLE similar approach to normalize data, so that we normalized the response by median level of controls which their quality is acceptable. Then we used a unified trapezoid approach to compute area under the curve (AUC) for both CCLE and GDSC. We also applied some quality controls to discard abnormal dose response curves. In a standard dose-response curve one expects that with larger concentration of drug cell viability would either remain stable or decrease. However, a small set of curves may not fit these assumptions due to experimental artifacts or normalization issues .e.g. cells do not grow well or were seeded too low. We

Detection of noisy experiments

The drug response curve of treatment of HCC-187 cell lines with Lapatinib was identified as noisy given that cumulative sum of delta values are equal to 63.94 which is greater than the considered threshold (2=50). However, this noisiness was likely to stem from a few points out of 18 and did not substantially affect drug sensitivity measurements, AAC of the drug response curve initially was 0.46 and was updated to 0.46 or 0.51 when two pairs of noisy measurements (2,5 or 2,9) were discarded. Similarly, sensitivity measurements of MDA-MB-157, MDA-MB-134, MFM-223, HCC-1187, HCC-202, ZR-75-1 and HCC-141 to AZD6244 were identified as noisy due to their cumulative sum greater than threshold (63.50, 64.13, 74.64, 51.05, 57.23, 72.80 and 74.39 respectively).

MDA-MB-157: AAC=0.25 with removing 18th point curve turns to be acceptable while AAC will remain similar to the original one (0.25).

MDA-MB-134: AAC=0.28 with removing 18th point curve turns to be acceptable while AAC will remain similar to the original one (0.30).

MFM-223: AAC=0.26 with removing both 17th and 18th points curve turns to be acceptable while AAC will remain similar to the original one (0.28).

HCC-1187: AAC=0.53 with removing any of 8th or 10th points curve turns to be acceptable while AAC will remain similar to the original one (0.53 or 0.56).

HCC202: AAC=0.27 with removing 18th point curve turns to be acceptable while AAC will remain similar to the original one (0.26).

ZR-75-1: AAC=0.22 with removing 18th point curve turns to be acceptable while AAC will remain similar to the original one (0.23).

HCC1419: AAC=0.25 with removing 18th point curve turns to be acceptable while AAC will remain similar to the original one (0.26).

Concentration range

Maximum concentration used for AZD6244 in CCLE, GDSC and GRAY datasets were 8 uM, 4 uM and 50 uM respectively. However, the applied dosage of drug in these datasets was not sufficiently large in these datasets which yields to not catching IC50 in 93% (29 of 31), 97% (37 of 38) and 83% (64 of 77) of experiments respectively. Therefore for our in vitro validation experiments we used a greater maximum concentration of AZD6244 to treat cells. We designed our experiments in two settings, in one setting we used a maximum concentration of 1000 uM in 3-fold change of drug dosage in 18 dilutions and in the second one we treated cells with a maximum concentration of 259 uM in 2-fold change of drug dosage in 9 dilutions. The first setting enabled us to catch the IC50 in 61% (8 of 13) of cases while the in second setting we were not still able to catch the IC50 for 92% (12 of 13) of cases. So we relied on the first setting for the rest of the analyses.

Research replicability

The code and documentation are available from the RNASeqDrug GitHub repository.

Acronyms

ABC	Area between the curves
AE	ArrayExpress by the European Bioinformatics Institute
AUC	Area under the dose response curve
AAC	Area above the dose response curve
CCLE	The Cancer Cell Line Encyclopedia initiated by the Broad Institute of MIT and Harvard
CGHub	The Cancer Genomics Hub from the University of California Santa Cruz and the US National Cancer [illegible]
CGP	The Cancer Genome Project by the Wellcome Trust Sanger Institute
CMAP	Connectivity Map by the Broad Institute
COSMIC	Catalogue of Somatic Mutations in Cancer by the Wellcome Trust Sanger Institute
CRAMERV	Carmer's V
DXY	Somers' Dxy rank correlation
GDSC	The Cancer Genome Project initiated by the Wellcome Trust Sanger Institute
IC_{50}	Concentration at which the drug inhibited 50% of the maximum cellular growth
INFORM	Informedness
MCC	Matthews correlation coefficient
PCC	Pearson product-moment correlation coefficient
QC	Quality control
RMA	Robust multi-array normalization
SCC	Spearman rank correlation coefficient
SNP	Single nucleotide polymorphism

Supplementary References

[1] Huixiao Hong, Lei Xu, Jie Liu, Wendell D Jones, Zhenqiang Su, Baitang Ning, Roger Perkins, Weigong Ge, Kelci Miclaus, Li Zhang, Kyunghee Park, Bridgett Green, Tao Han, Hong Fang, Christophe G Lambert, Silvia C Vega, Simon M Lin, Nadereh Jafari, Wendy Czika, Russell D Wolfinger, Federico Goodsaid, Weida Tong, and Leming Shi. Technical Reproducibility of Genotyping SNP Arrays Used in Genome-Wide Association Studies. *PloS one*, 7(9):e44483, September 2012.

[2] Christiaan Klijn, Steffen Durinck, Eric W Stawiski, Peter M Haverty, Zhaoshi Jiang, Hanbin Liu, Jeremiah Degenhardt, Oleg Mayba, Florian Gnad, Jinfeng Liu, Gregoire Pau, Jens Reeder, Yi Cao, Kiran Mukhyala, Suresh K Selvaraj, Mamie Yu, Gregory J Zynda, Matthew J Brauer, Thomas D Wu, Robert C Gentleman, Gerard Manning, Robert L Yauch, Richard Bourgon, David Stokoe, Zora Modrusan, Richard M Neve, Frederic J de Sauvage, Jeffrey Settleman, Somasekar Seshagiri, and Zemin Zhang. A comprehensive transcriptional portrait of human cancer cell lines. *Nat Biotech*, 33 (3):306–312, March 2015.

Supplementary Tables

Dataset	Compounds	Cell lines	Tissue types	RNA-seq availability	Assay
CCLE	24	1061	24	TRUE	CellTiter-Glo
GDSC	139	1124	36	FALSE	Syto60
gSCI	16	410	24	TRUE	CellTiter-Glo
GRAY	90	84	1	TRUE	CellTiter-Glo
UHN	4	84	1	TRUE	CellTiter-Glo

Supplementary Table 1: Collection of pharmacogenomic datasets and their characteristics (including number of cell lines, tissue types, RNA-seq availability, pharmacological assay).

Compound	Training	Pre-validation Pan-Cancer	Training Breast Specific	Pre-validation Breast	Final validation
17-AAG	591	-	275	6	-
AZD6244	150	-	65	12	1
Crizotinib	11	3	3	0	-
Erlotinib	80	48	37	11	8
lapatinib	57	32	27	5	1
Nutlin-3	4	-	2	1	-
paclitaxel	290	22	137	35	6
PD-0325901	1333	517	-	-	-
Sorafenib	38	-	17	0	-

Supplementary Table 2: Number of significant isoformic biomarkers is identified in (A) CCLE and GDSC (training sets); (B) gCSI (pre-validation pan-cancer) (C) GRAY (pre-validation breast) and (D) UHN (final validation).

Cell line	Doubling time
600MPE	0.55
AU655	1.49
BT-20	1.81
BT-474	2.66
BT-549	1.97
CAL-120	1.15
CAL-148	1.75
CAL-51	0.8
CAMA-1	1.64
EVSA-T	0.86
HBL-100	1.05
HCC1143	2.02
HCC1187	2.47
HCC1395	2.29
HCC1419	3.76
HCC1806	1.73
HCC1937	2.4
HCC1954	1.96
HCC202	2.78
HCC2185	2.5
HCC3153	2.8
HCC38	2.16
HCC70	2.46
HDQ-P1	2.13
Hs-578-T	1.29
JIMT-1	2.02
KPL-1	1.32
LY2	1.82
MCF7	1.53
MDA-MB-157	2.13
MDA-MB-231	1.01
MDA-MB-330	3.19
MDA-MB-361	1.98
MDA-MB-436	1.49
MDA-MB-468	1.61
MFM-223	1.74
OCUB-M	1.65
SK-BR-3	2.05
SW 527	1.04
T47D	1.81
UACC-893	5.48
ZR-75-1	2.53

Supplementary Table 3: Population doubling time of the cell lines used in the final validation (UHN).

drug	gene	isoform	Training cindex	Breast cindex	Training fdr	Final validation cindex
AZD6244	IGF2BP2	ENST00000346192	0.62	0.74	8.1E-06	0.68
Erlotinib	ITGB6	ENST00000283249	0.63	0.76	1.2E-06	0.89
Erlotinib	CDH3	ENST00000567674	0.61	0.67	4.3E-04	0.76
Erlotinib	GJB3	ENST00000373362	0.62	0.80	1.1E-06	0.74
Erlotinib	GSDMC	ENST00000619643	0.61	0.71	4.1E-04	0.68
Erlotinib	MST1R	ENST00000344206	0.61	0.71	5.5E-05	0.64
Erlotinib	GPRIN2	ENST00000374317	0.61	0.57	1.6E-05	0.63
lapatinib	NECTIN4	ENST00000368012	0.64	0.64	2.2E-04	0.83
paclitaxel	KLHDC9	ENST00000490724	0.60	0.65	2.6E-03	0.79
paclitaxel	FAM174A	ENST00000312637	0.60	0.59	5.0E-03	0.77
paclitaxel	RPL12	ENST00000361436	0.61	0.67	6.8E-10	0.74
paclitaxel	SNRPB	ENST00000474384	0.61	0.55	9.8E-09	0.70
paclitaxel	RPSA	ENST00000443003	0.60	0.58	1.3E-05	0.70
paclitaxel	SCUBE2	ENST00000519535	0.60	0.59	8.5E-05	0.69
paclitaxel	MAGED2	ENST00000375058	0.61	0.64	5.6E-03	0.68
paclitaxel	EPB41L4A	ENST00000305368	0.60	0.61	4.0E-03	0.62

Supplementary Table 4: List of novel isoformic biomarkers in the final validation set (UHN) along with their predictive value (concordance index) and significance in training set. The concordance index of biomarkers specific to breast cancer cell lines in the training sets is also reported. Half the biomarkers yielded higher concordance index in breast cancer cell lines compared to pan-cancer estimates (training cindex), likely due to the reduced molecular heterogeneity in specific tissue type. The biomarkers are ordered by their predictive value (concordance index) in validation model which is reported as the last column.

drug	gene	type	gene cindex	gene pvalue	isoform	isoform cindex	isoform pvalue	null model cindex
17-AAG	NQO1	expression	0.69	2.0E-18	ENST00000320623	0.68	1.4E-17	0.60
PD-0325901	BRAF	mutation	0.64	1.2E-02				0.62
AZD6244	BRAF	mutation	0.61	4.4E-01				0.61
TAE684	ALK	expression	0.58	1.0E+00	ENST00000431873	0.59	1.0E+00	0.59
Crizotinib	MET	amplification	0.57	7.3E-01				0.57
PLX4720	BRAF	mutation	0.61	7.8E-15				0.58
Nutlin-3	MDM2	expression	0.56	1.2E-14	ENST00000539479	0.55	5.7E-13	0.53
lapatinib	ERBB2	expression	0.64	2.8E-01	ENST00000582818	0.61	1.0E+00	0.62
lapatinib	ERBB2	amplification	0.61	1.0E+00				0.62
PHA-665752	MET	amplification	0.54	9.9E-01				0.55
Erlotinib	EGFR	mutation	0.60	1.0E+00				0.61
Sorafenib	FLT3	mutation	0.54	2.4E-01				0.54

Supplementary Table 5: List of known associations with their effect size and significance in training set. The null model includes tissue as sole predictor for drug sensitivity, while the gene or isoform models includes tissue and expression as predictors.

Supplementary Figures

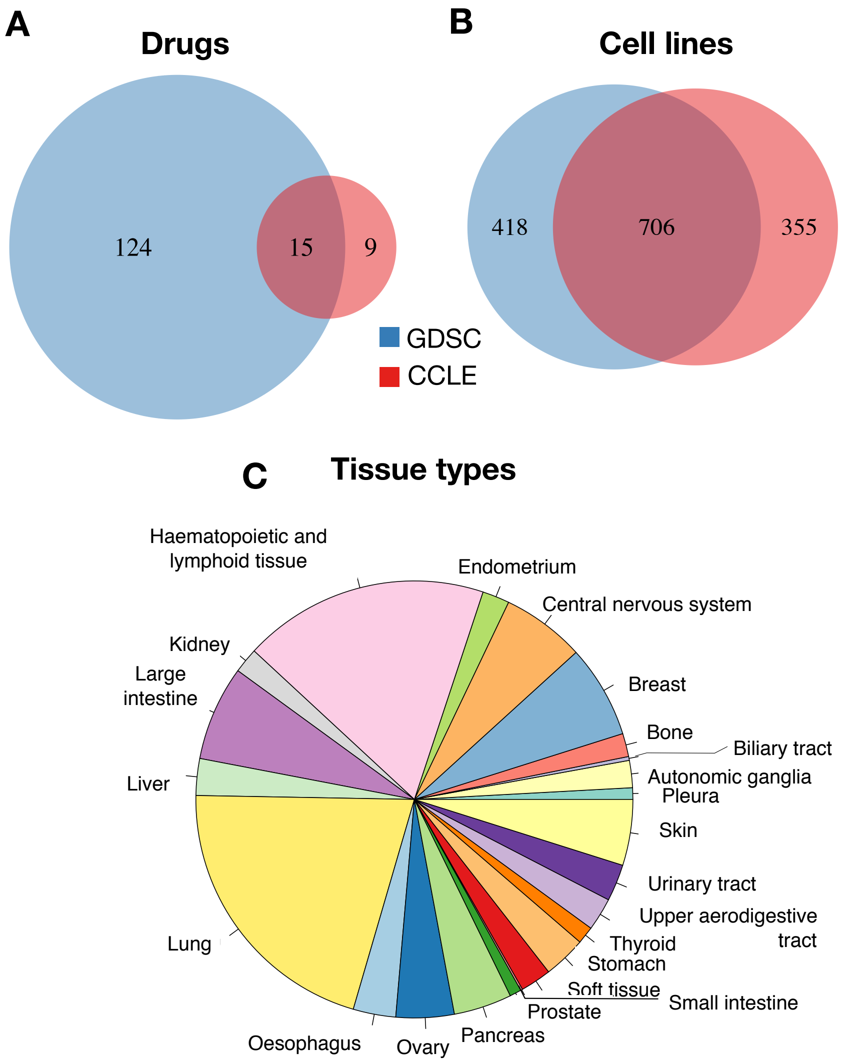

Supplementary Figure 1: Intersection between GDSC and CCLE. Overlap of (A) drugs, (B) cell lines and (C) tissue types.

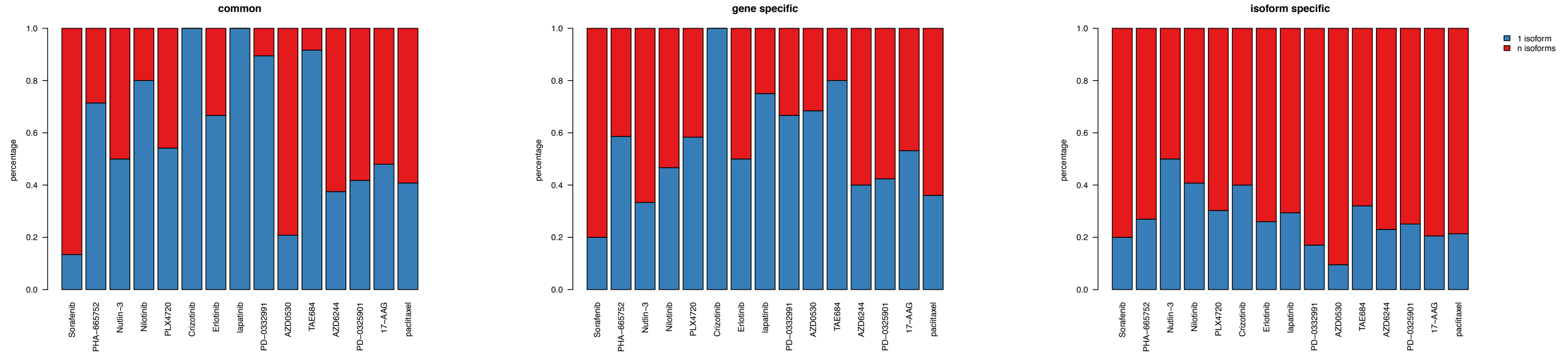

Supplementary Figure 2: The significant associations in training phase are grouped into three classes, isoform specific, gene specific, and common containing the biomarkers significant at both the gene and isoform levels. Each plot shows the ratio of biomarkers according to the number of alternatively spliced events in their corresponding genes. The ones with just one transcript are colored in red while the ones with more than one isoforms are shown in blue.

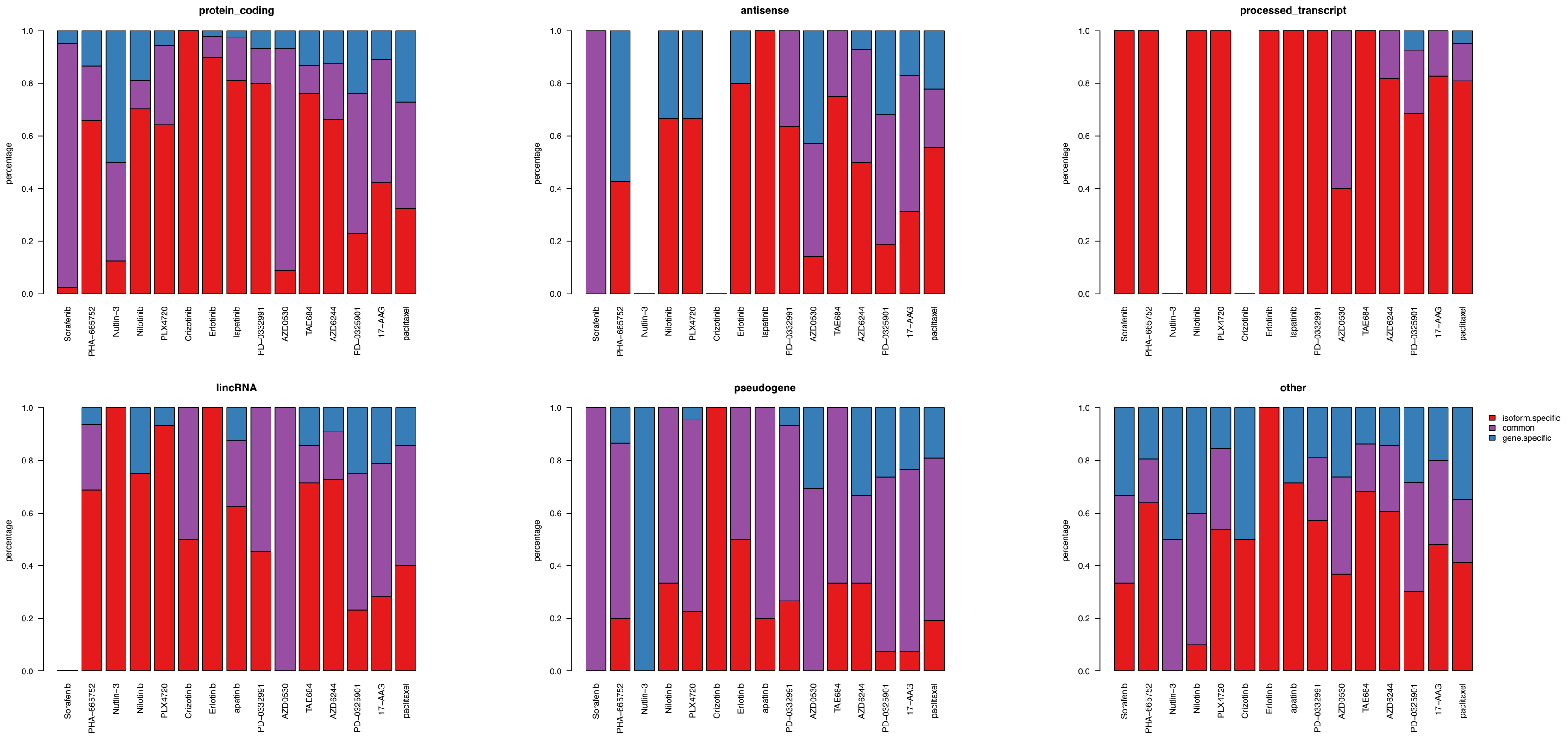

Supplementary Figure 3: The classification of significant associations in training phase according to their bio-types: protein coding, antisense, processed transcript, lincRNA and other.

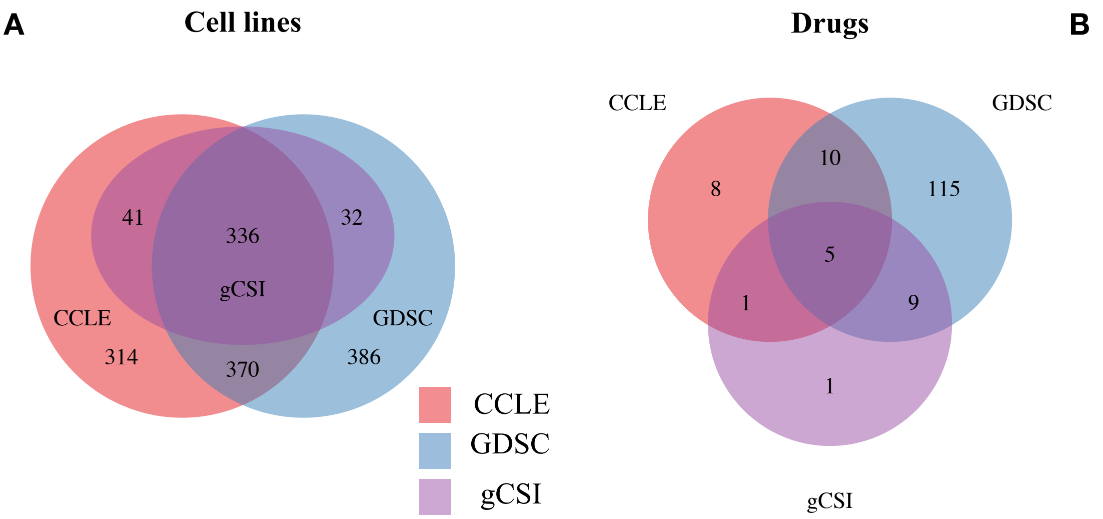

Supplementary Figure 4: Overlap of (A) cell lines and (B) cell lines between CCLE, GDSC and gCSI.

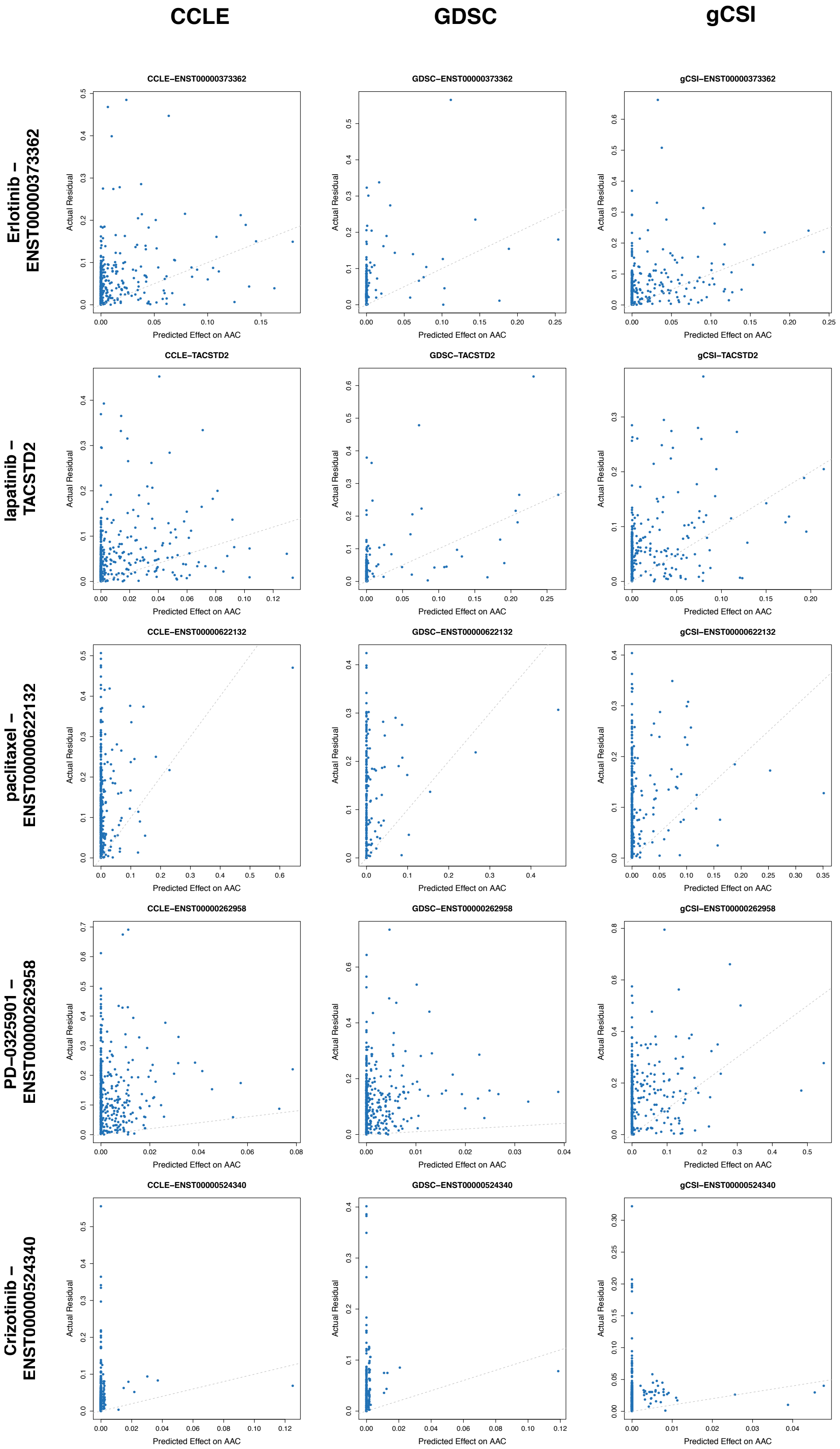

Supplementary Figure 5: The predicted change in AAC for top biomarker for each drug controlled for tissue vs the actual residuals of the predictions based on tissue terms only in gCSI.

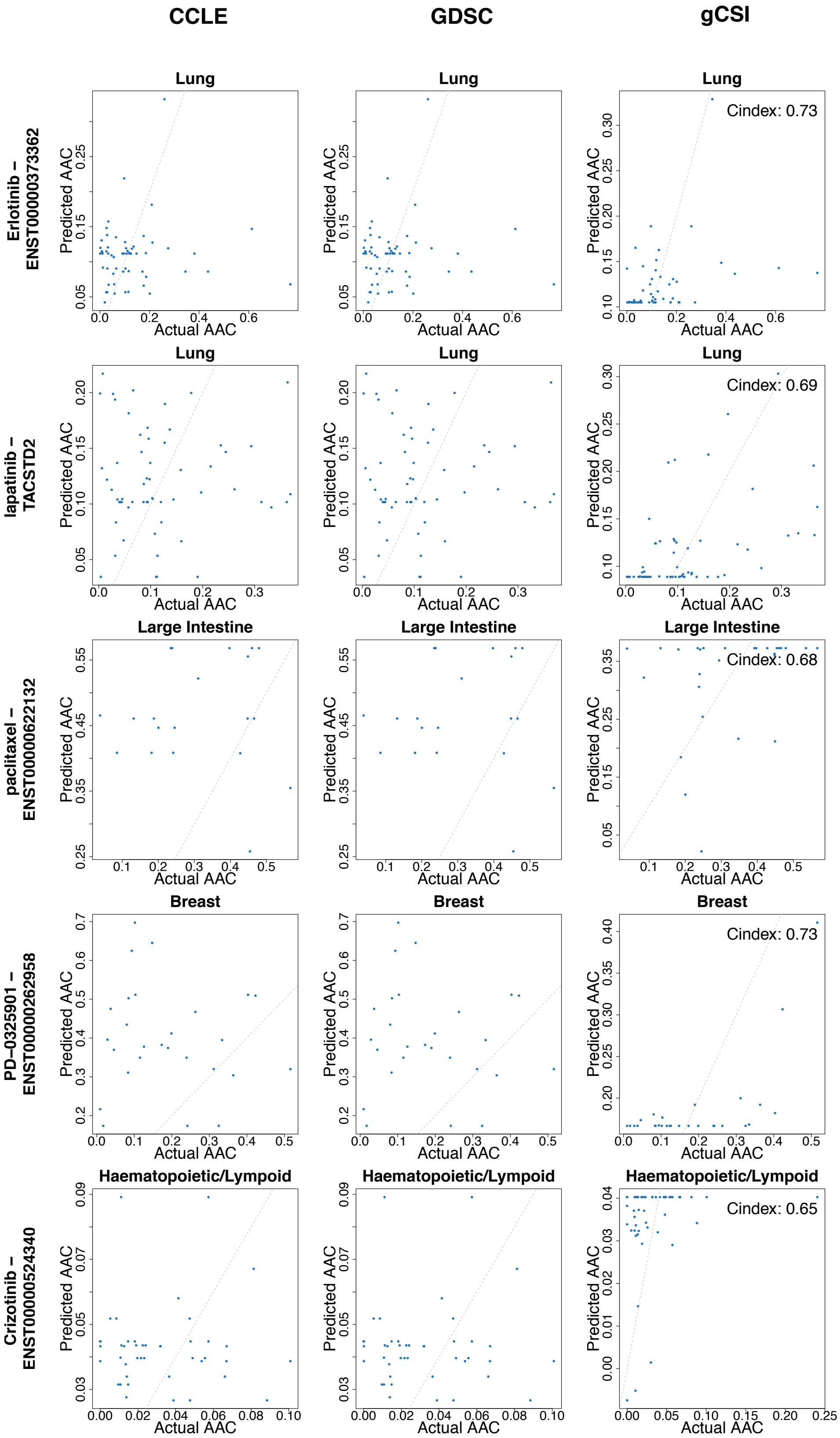

Supplementary Figure 6: The predicted vs actual AAC for each dataset and top biomarker for each drug, plotted for the tissue with top c-index in gCSI out of the largest 5 tissues (in gCSI).

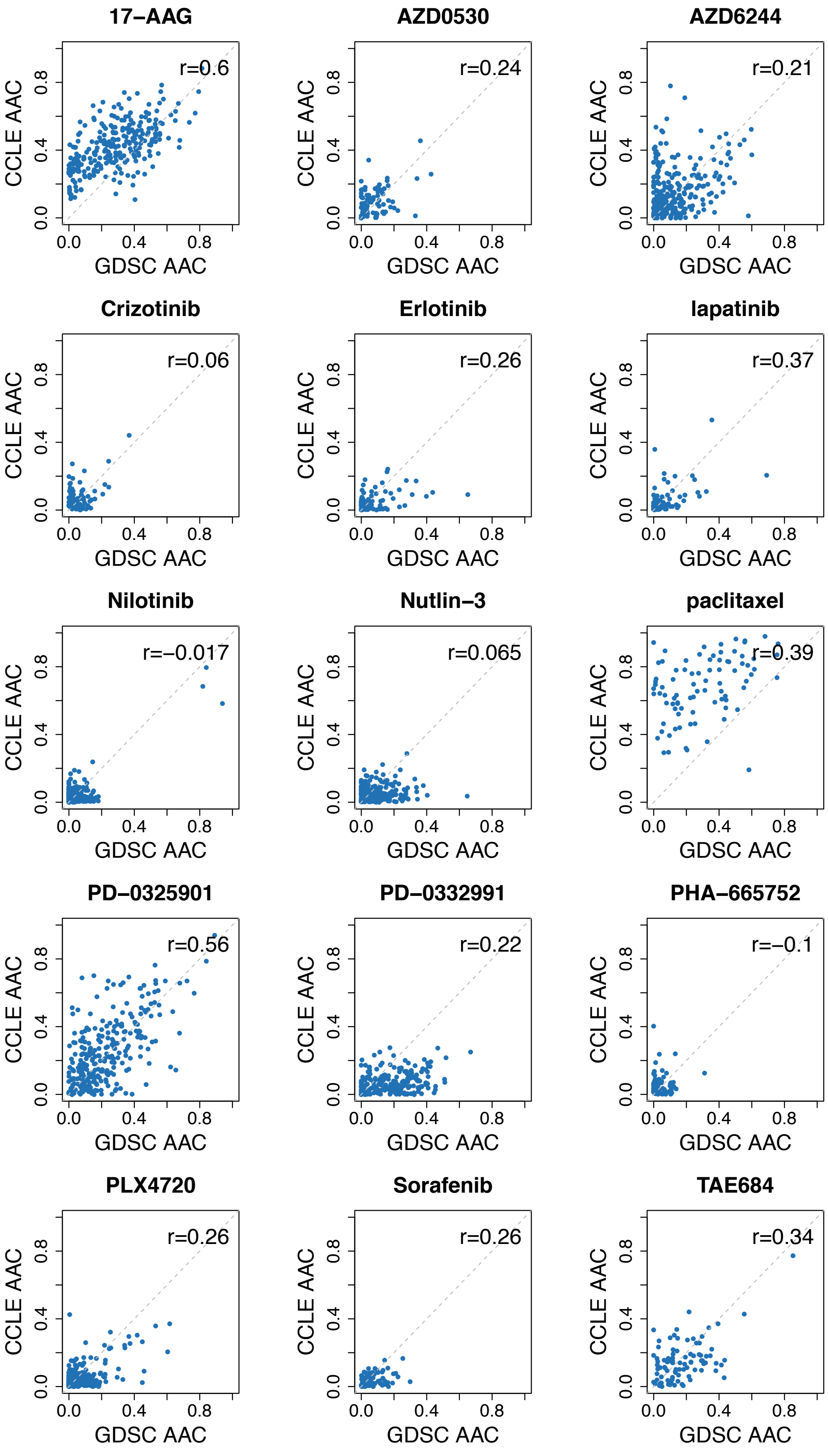

Supplementary Figure 7: Consistency in drug sensitivity data across CCLE and GDSC for drugs in common between datasets.

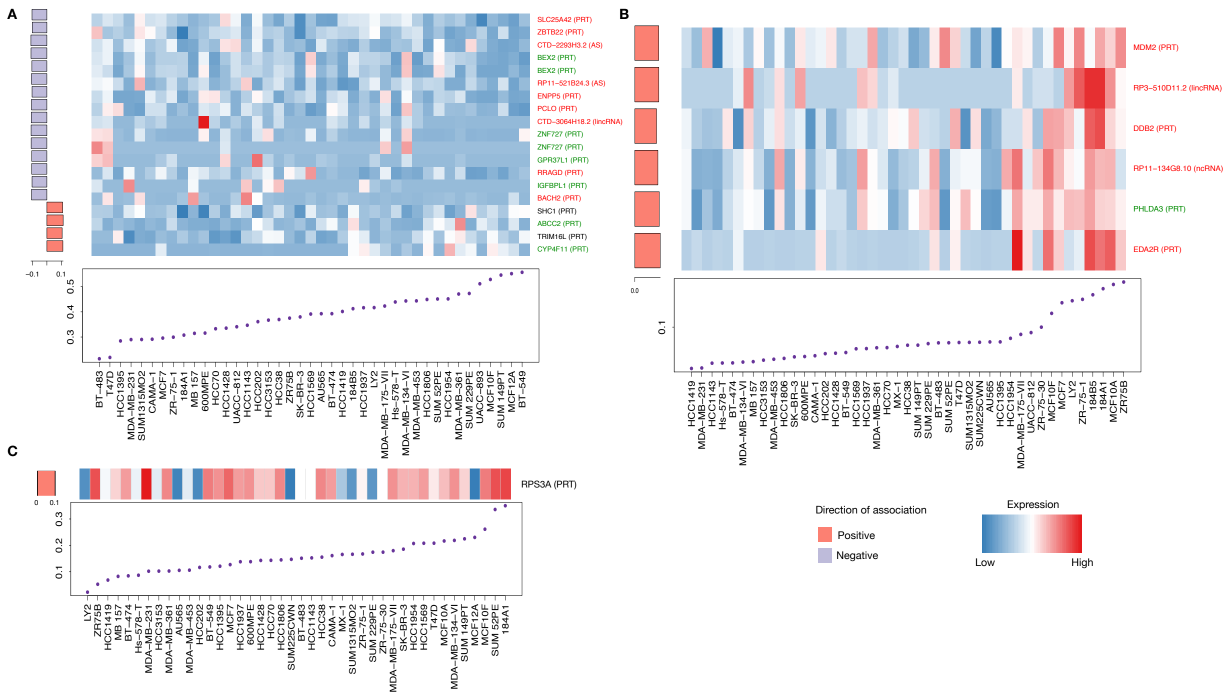

Supplementary Figure 8: Isoform-based biomarkers successfully pre-validated in the independent GRAY dataset for (A) Sorafenib (B) Nutlin-3 (C) 17-AAG. There was no biomarkers validated for crizotinib. Cell lines are ordered by their sensitivity to the drug of interest and their isoform expression is shown in the heatmap, with the drug sensitivity (AAC) plotted below. The left side barplot shows the predictive value of the associations between isoform expression and drug sensitivity as the concordance index multiplied by the sign of the coefficient in the corresponding regression model. Genes for which the candidate isoform is significantly more predictive than its corresponding overall gene expression values are represented in green, red when overall gene expression is more predictive than isoform, and black otherwise.

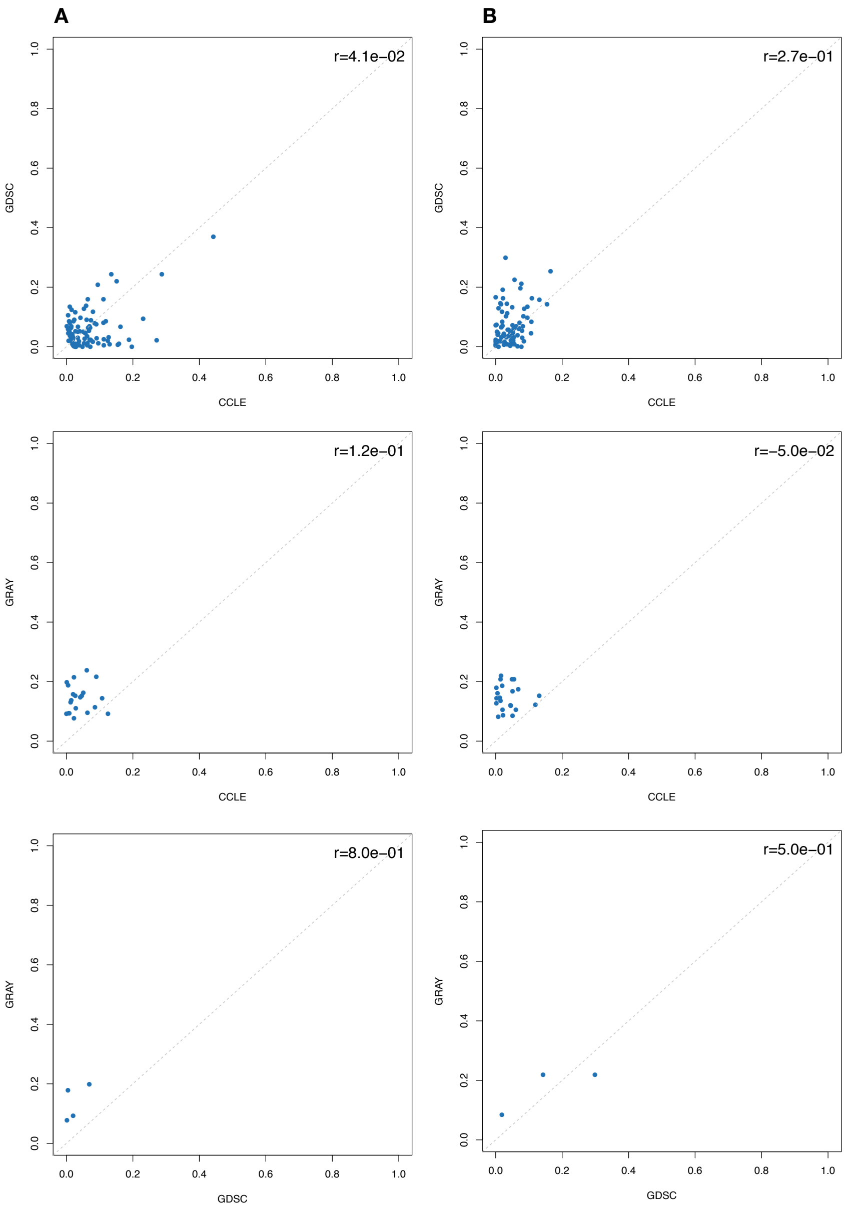

Supplementary Figure 9: Consistency in drug sensitivity data across CCLE, GDSC and GRAY for (A) crizotinib and (B) sorafenib.

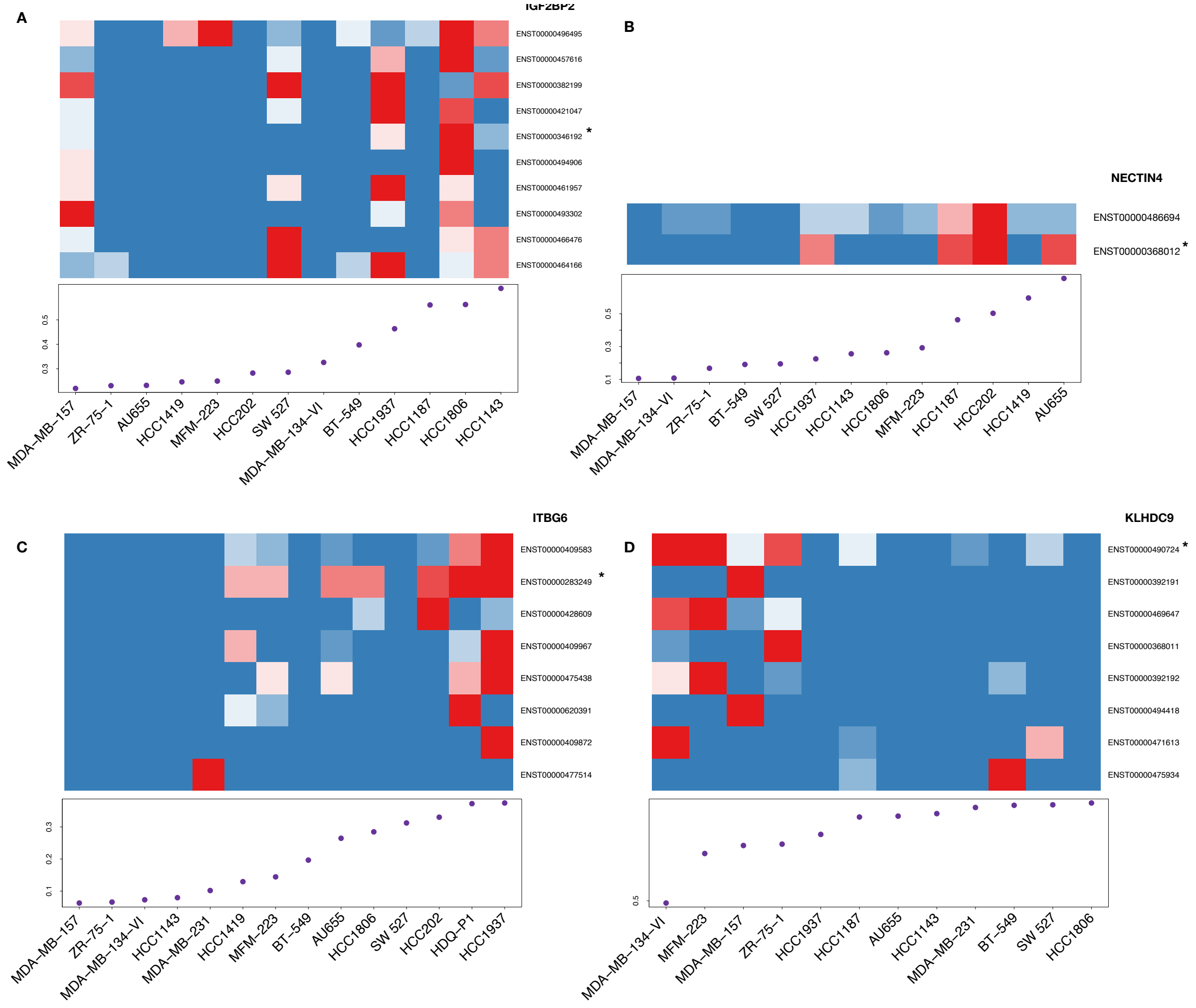

Supplementary Figure 10: Expression of all the isoforms for the selected biomarkers predictive of the response to (A) AZD6244 (B) lapatinib (C) erlotinib (D) paclitaxel.

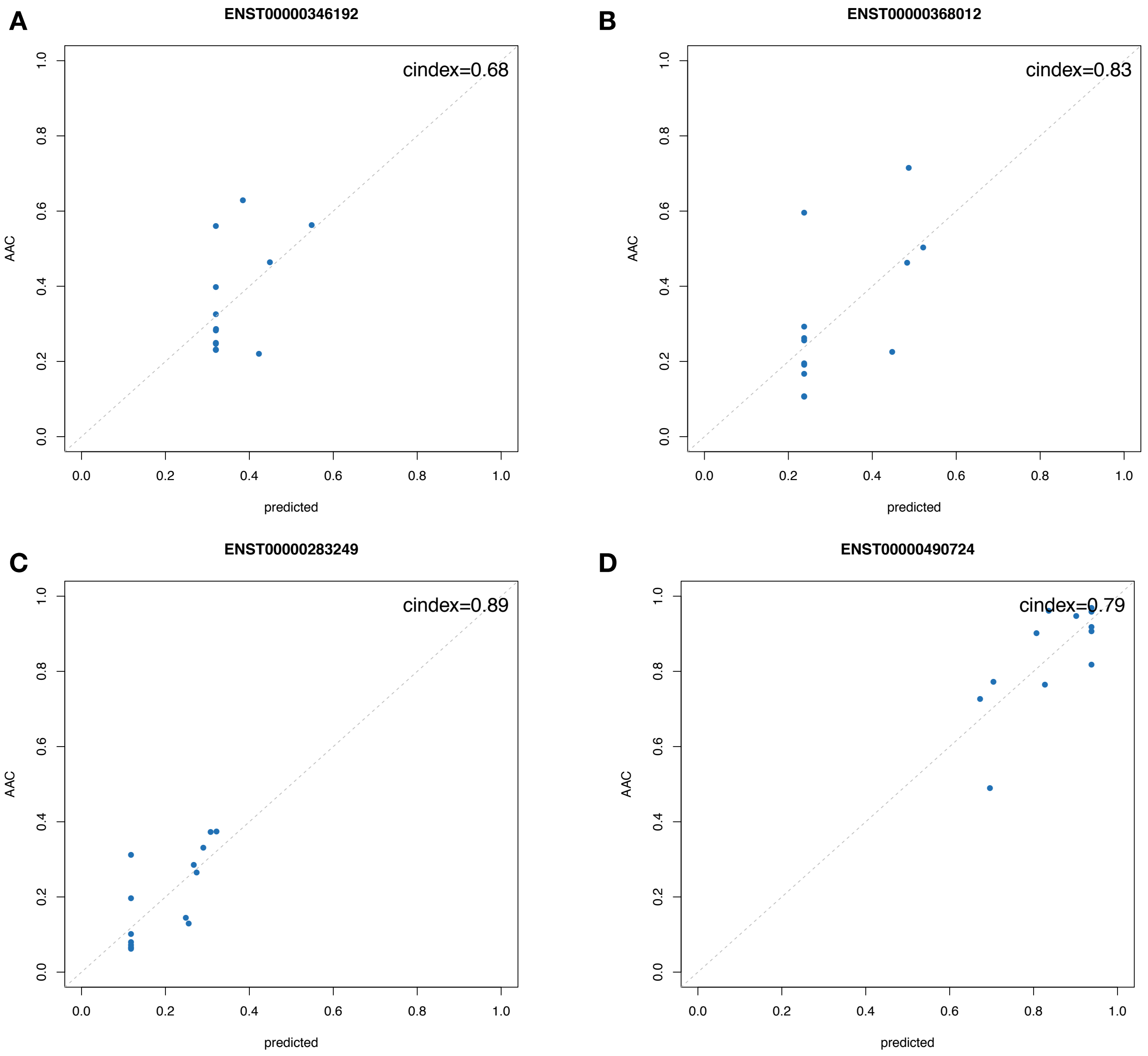

Supplementary Figure 11: The predicted AAC values versus the actual AAC values for the most significant validated biomarkers in UHN for (A) AZD6244 (B) lapatinib (C) erlotinib (D) paclitaxel.

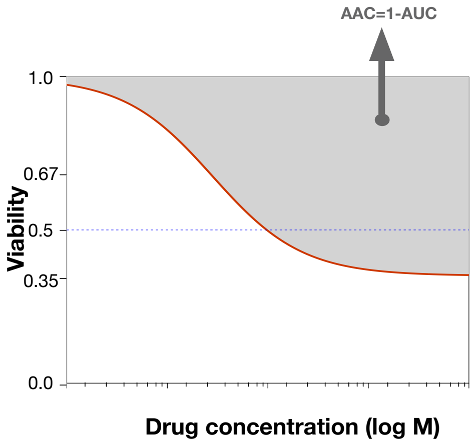

Supplementary Figure 12: Example of drug dose-response curve. AAC: area above the curve, AUC: area under the curve. The higher AAC is, the more sensitive is the cell line the drug of interest. The inverse is true for AUC.

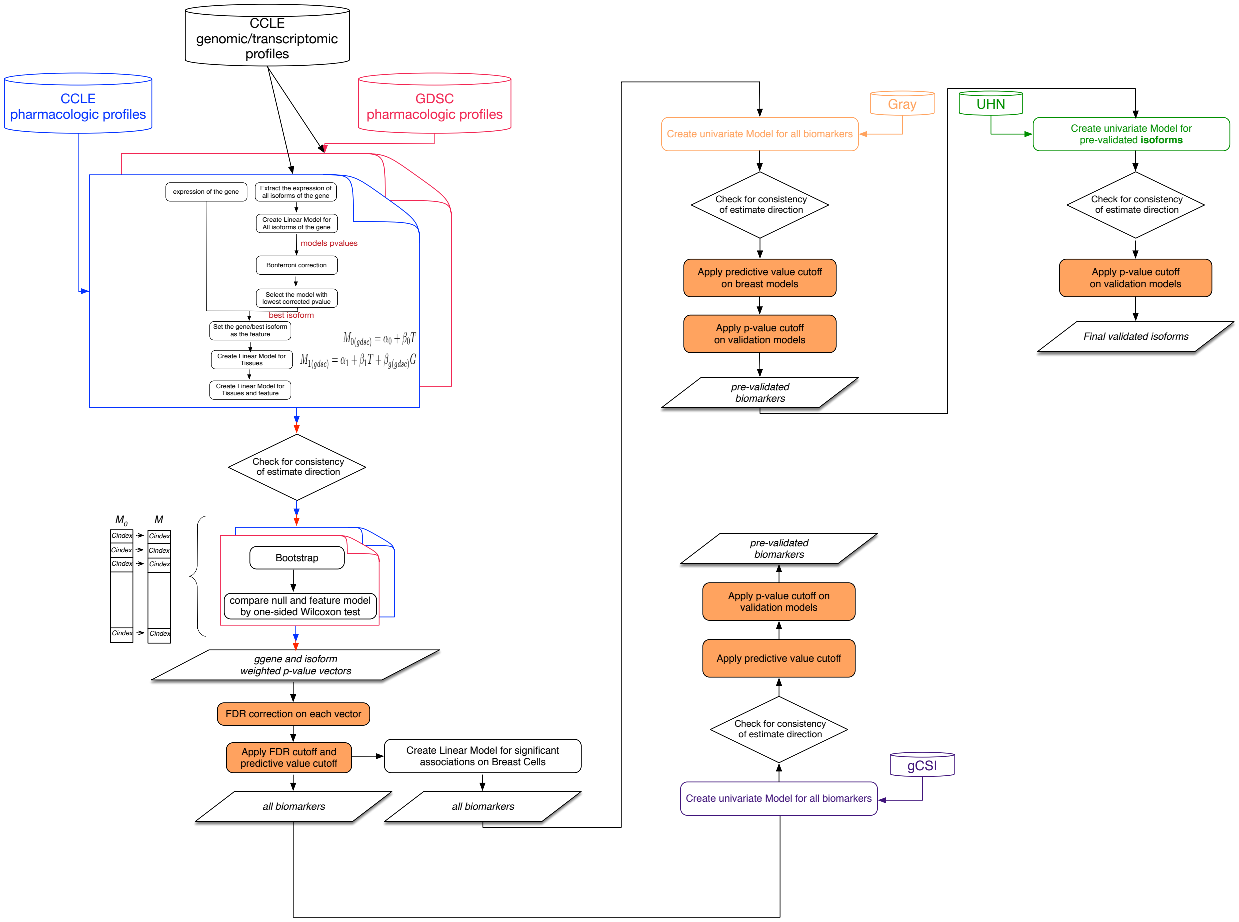

Supplementary Figure 13: Overview of the methodological approach used to identify and (pre-)validate the candidate biomarkers in vitro.

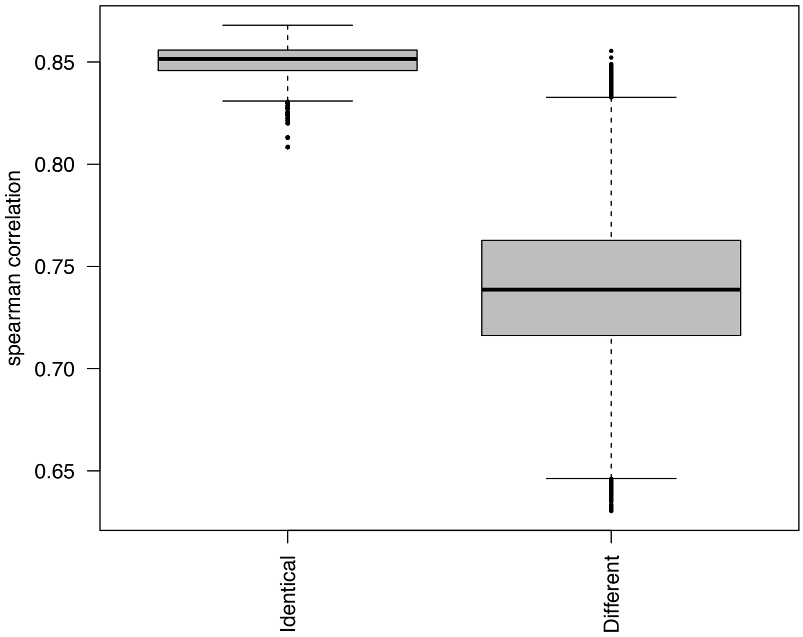

Supplementary Figure 14: Correlation between CCLE microarray and RNA-seq gene expression profiles.

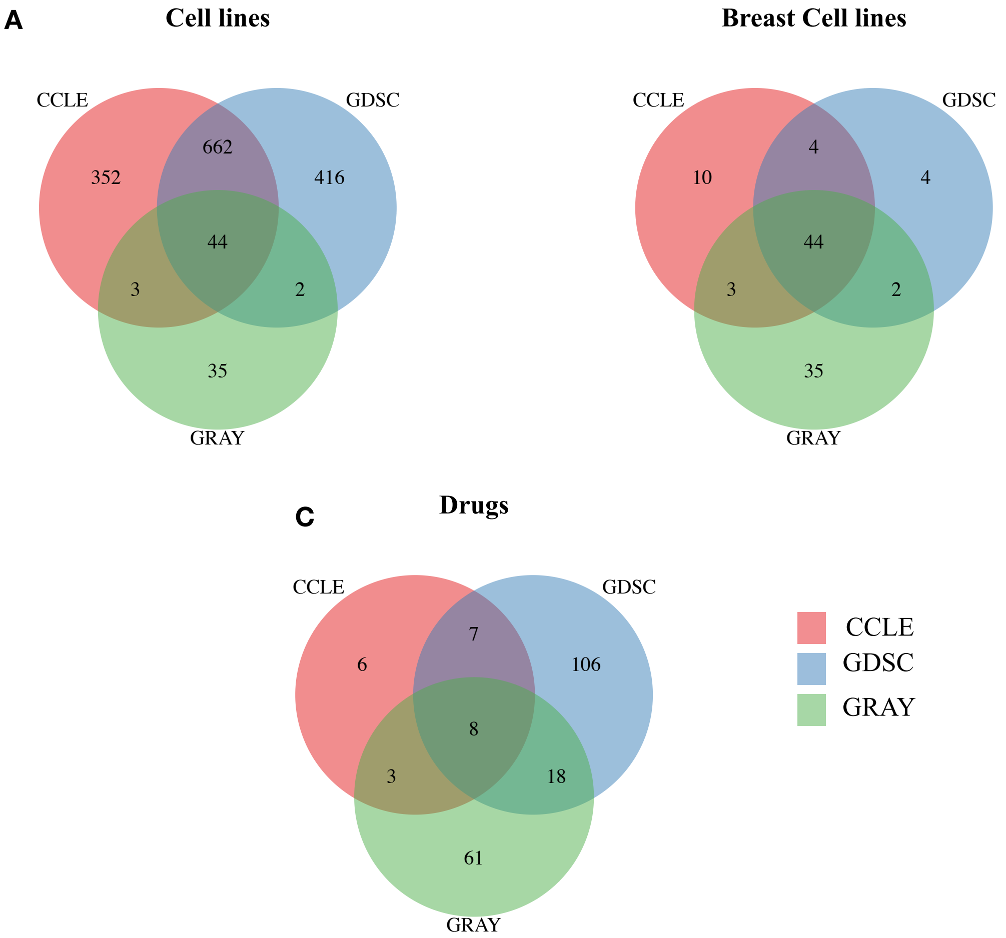

Supplementary Figure 15: Overlap of (A) cell lines, (B) breast cell lines and (C) drugs between CCLE, GDSC and GRAY.

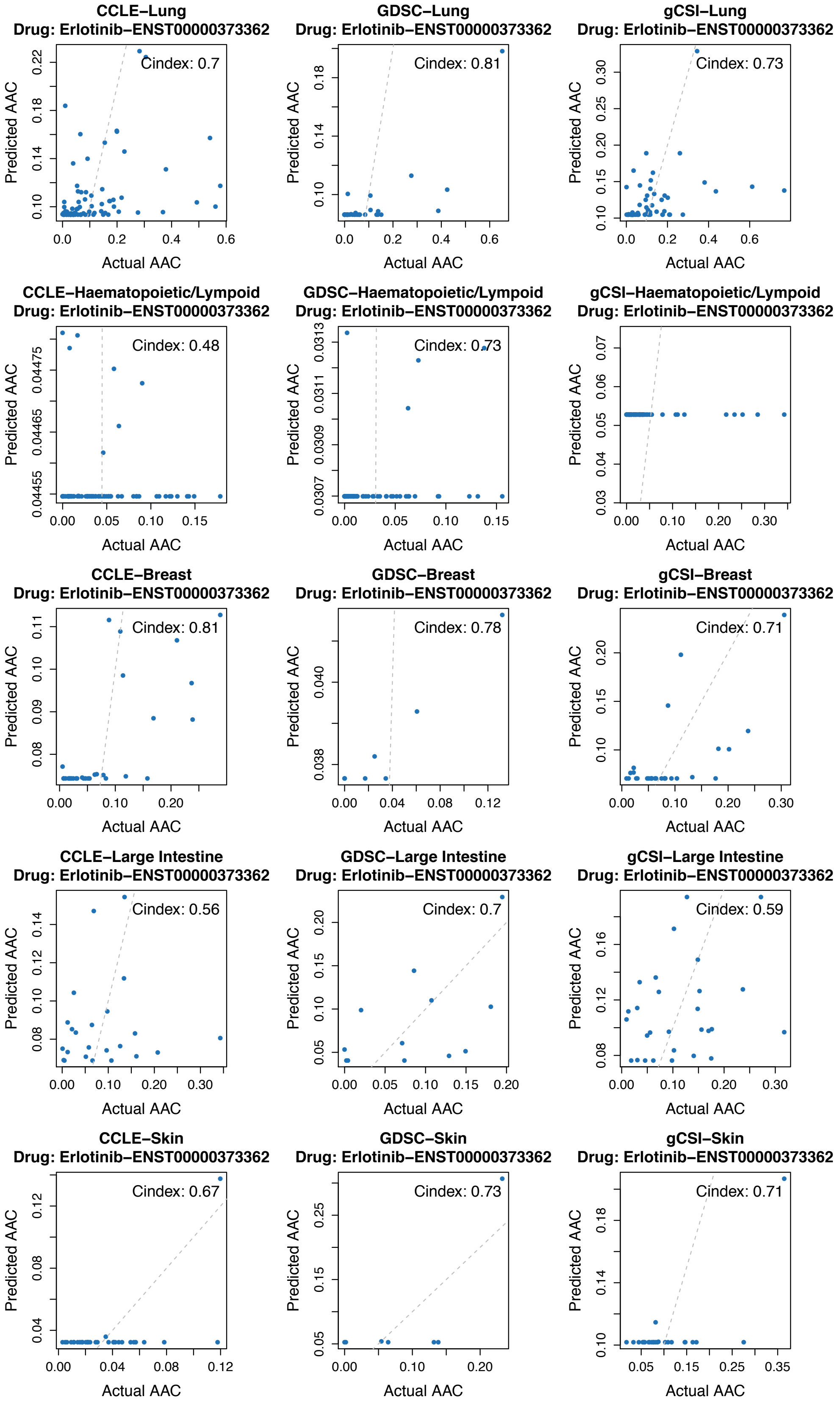

Supplementary Figure 16: Predicted versus actual AAC in each tissue type for the top biomarker associated with the drugs in common between gCSI, CCLE and GDSC.

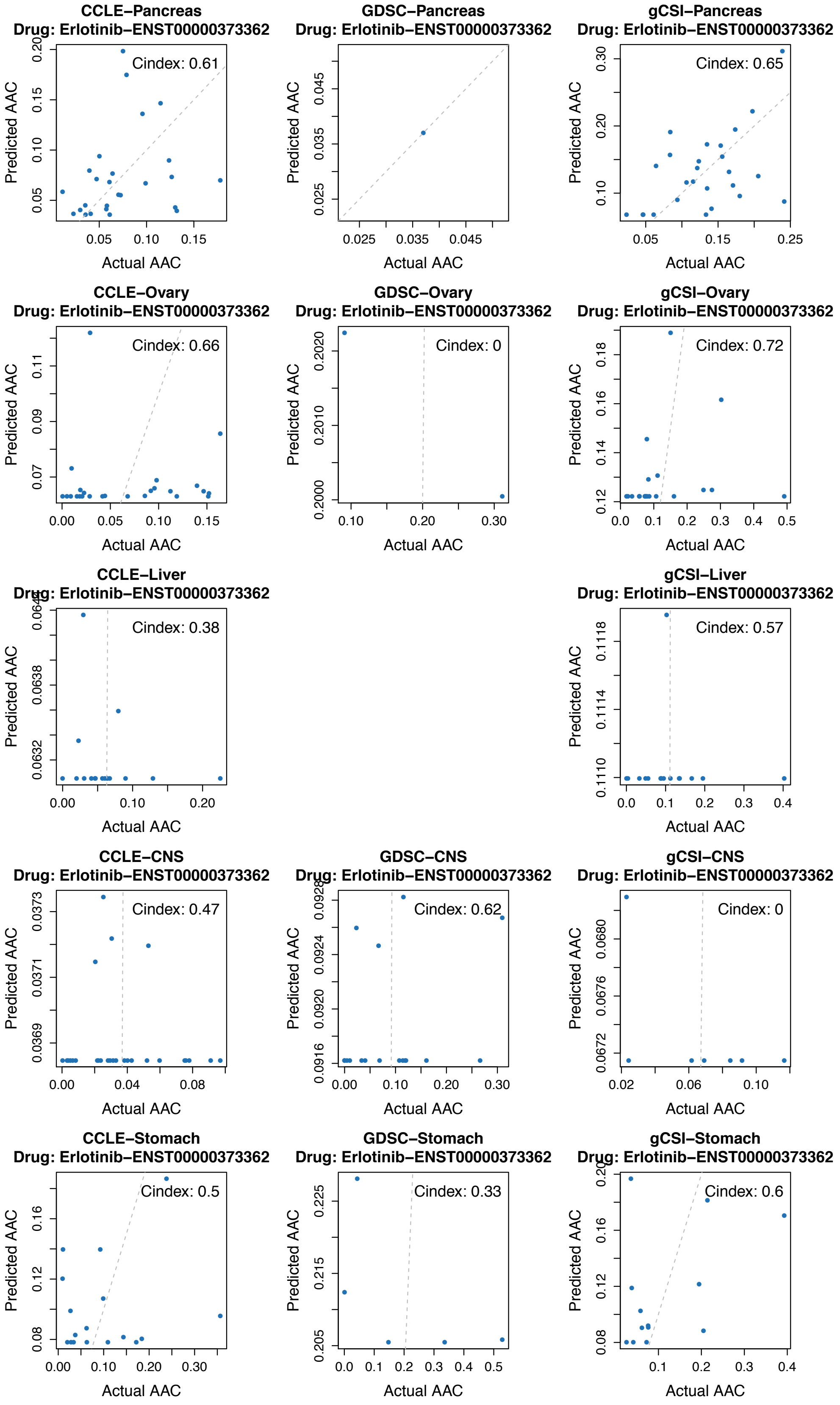

Supplementary Figure 16 (cont'd): Predicted versus actual AAC in each tissue type for the top biomarker associated with the drugs in common between gCSI, CCLE and GDSC.

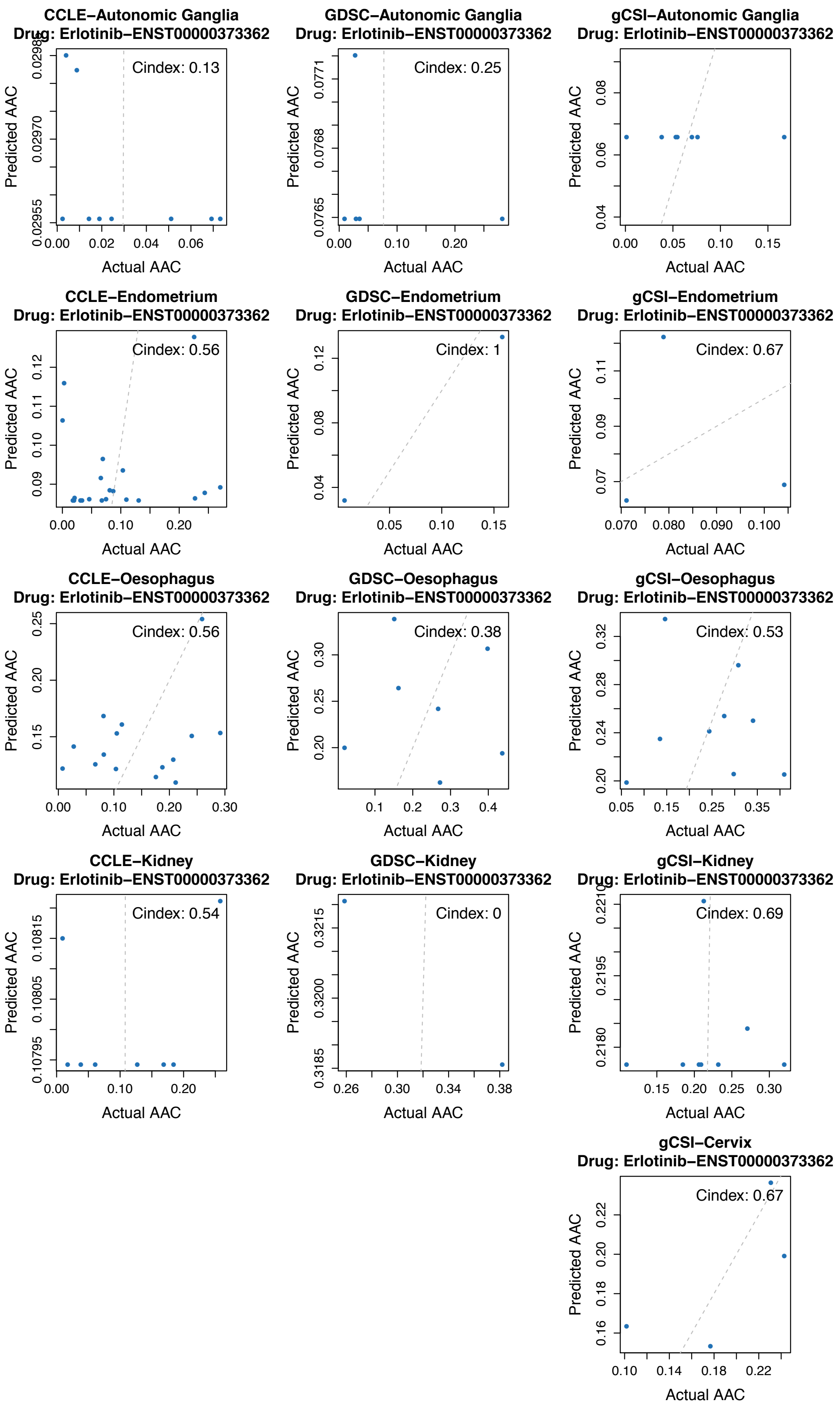

Supplementary Figure 16 (cont'd): Predicted versus actual AAC in each tissue type for the top biomarker associated with the drugs in common between gCSI, CCLE and GDSC.

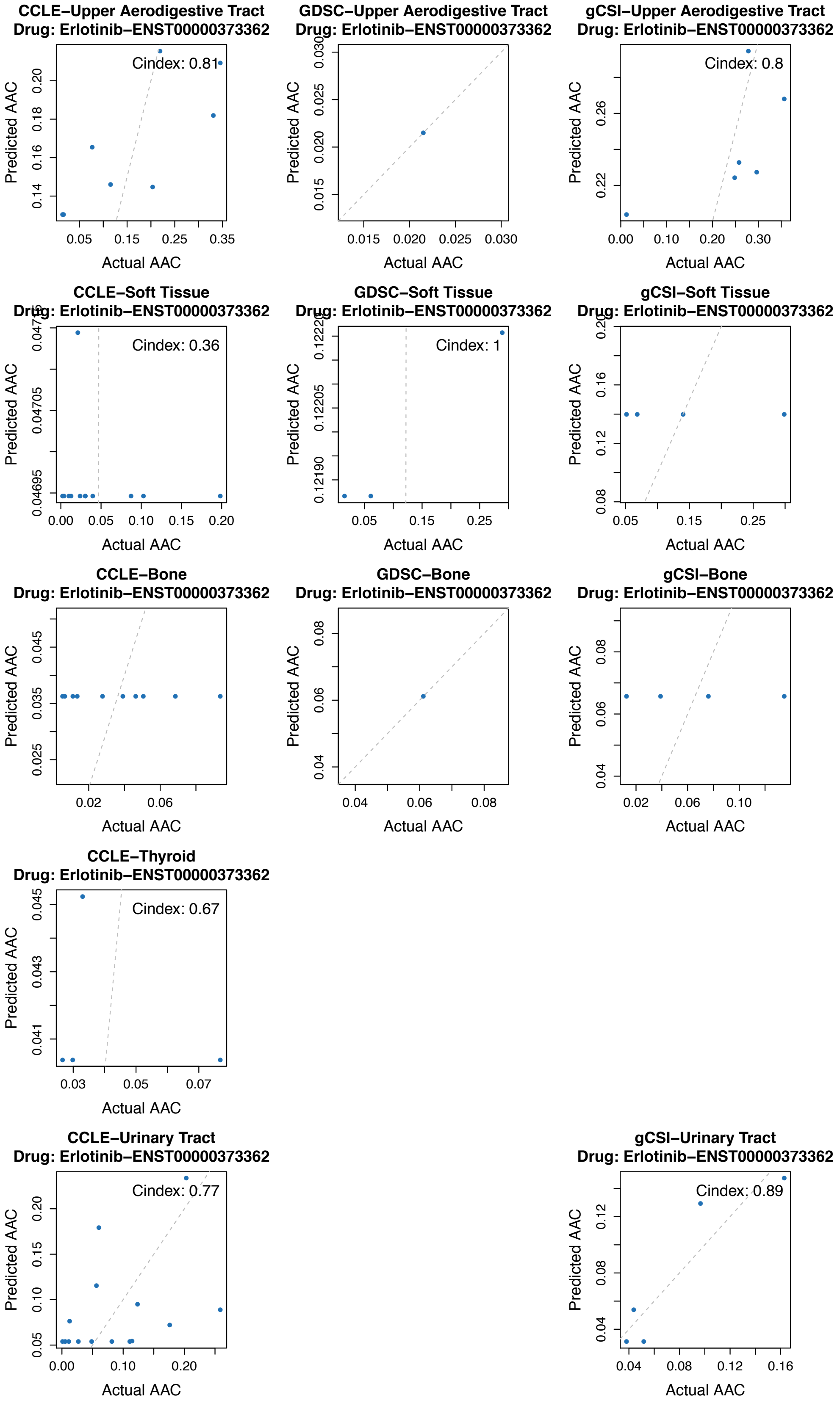

Supplementary Figure 16 (cont'd): Predicted versus actual AAC in each tissue type for the top biomarker associated with the drugs in common between gCSI, CCLE and GDSC.

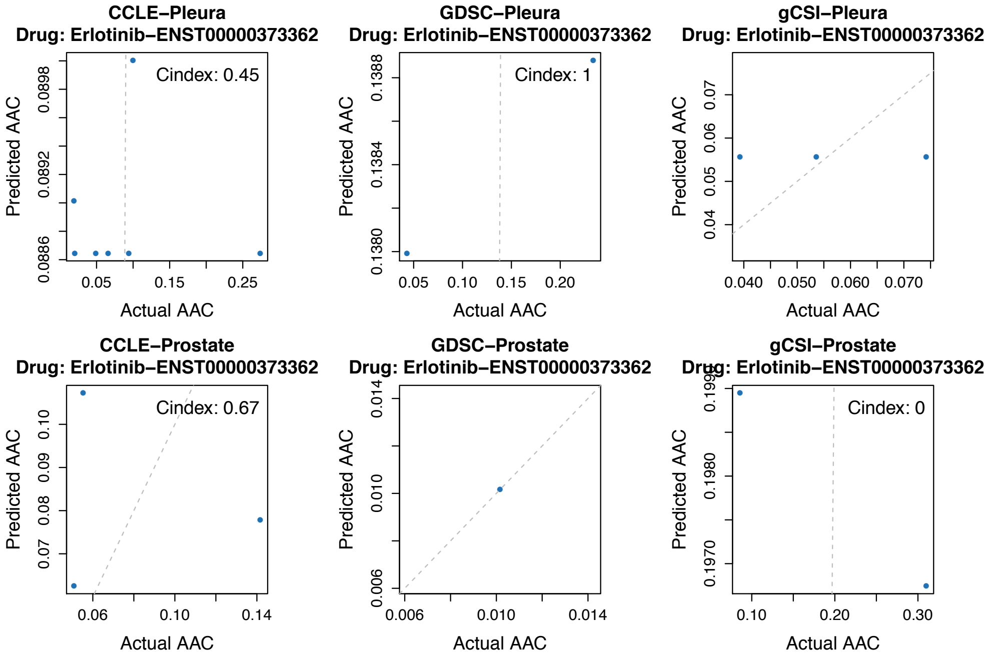

Supplementary Figure 16 (cont'd): Predicted versus actual AAC in each tissue type for the top biomarker associated with the drugs in common between gCSI, CCLE and GDSC.

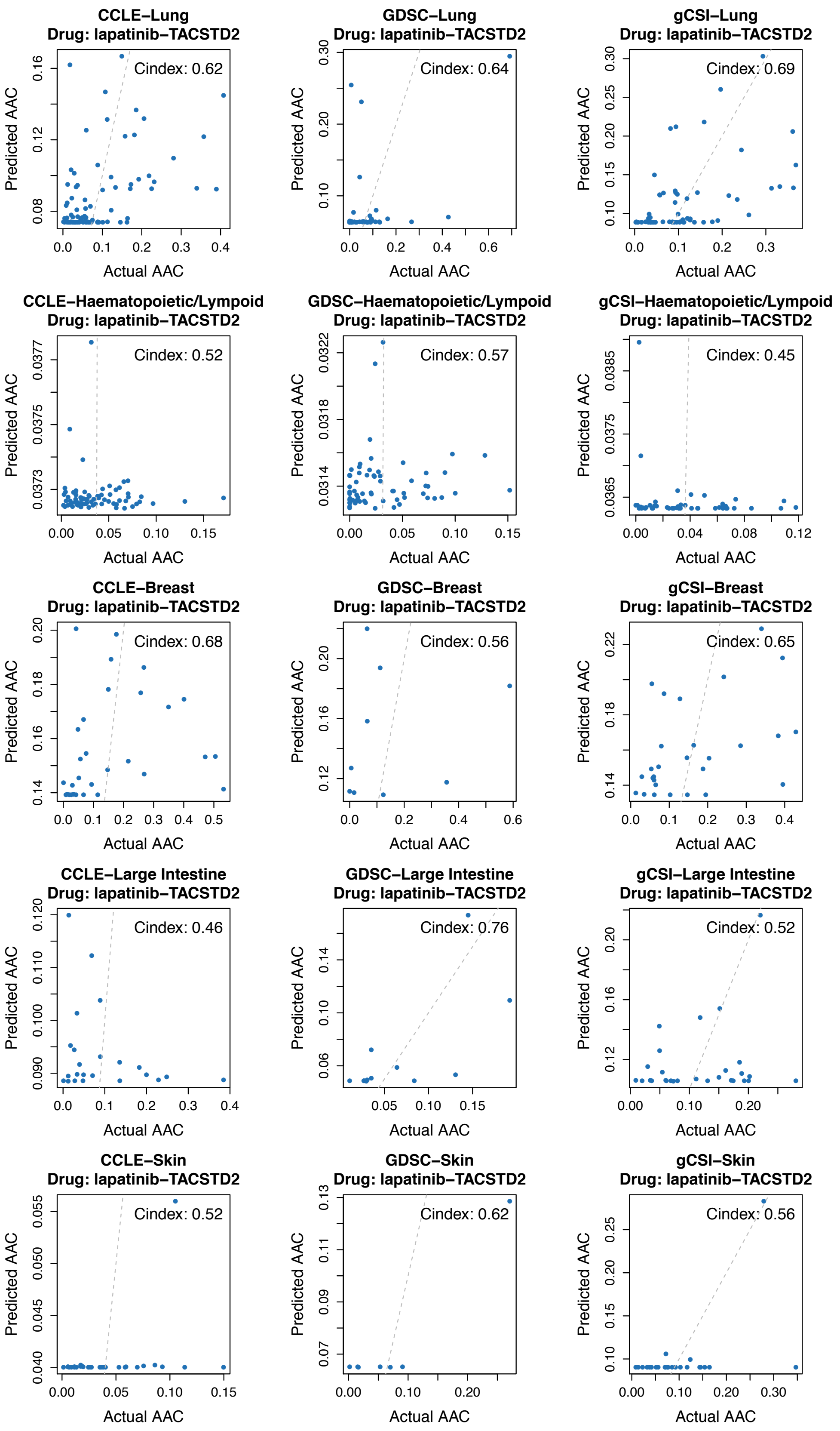

Supplementary Figure 16 (cont'd): Predicted versus actual AAC in each tissue type for the top biomarker associated with the drugs in common between gCSI, CCLE and GDSC.

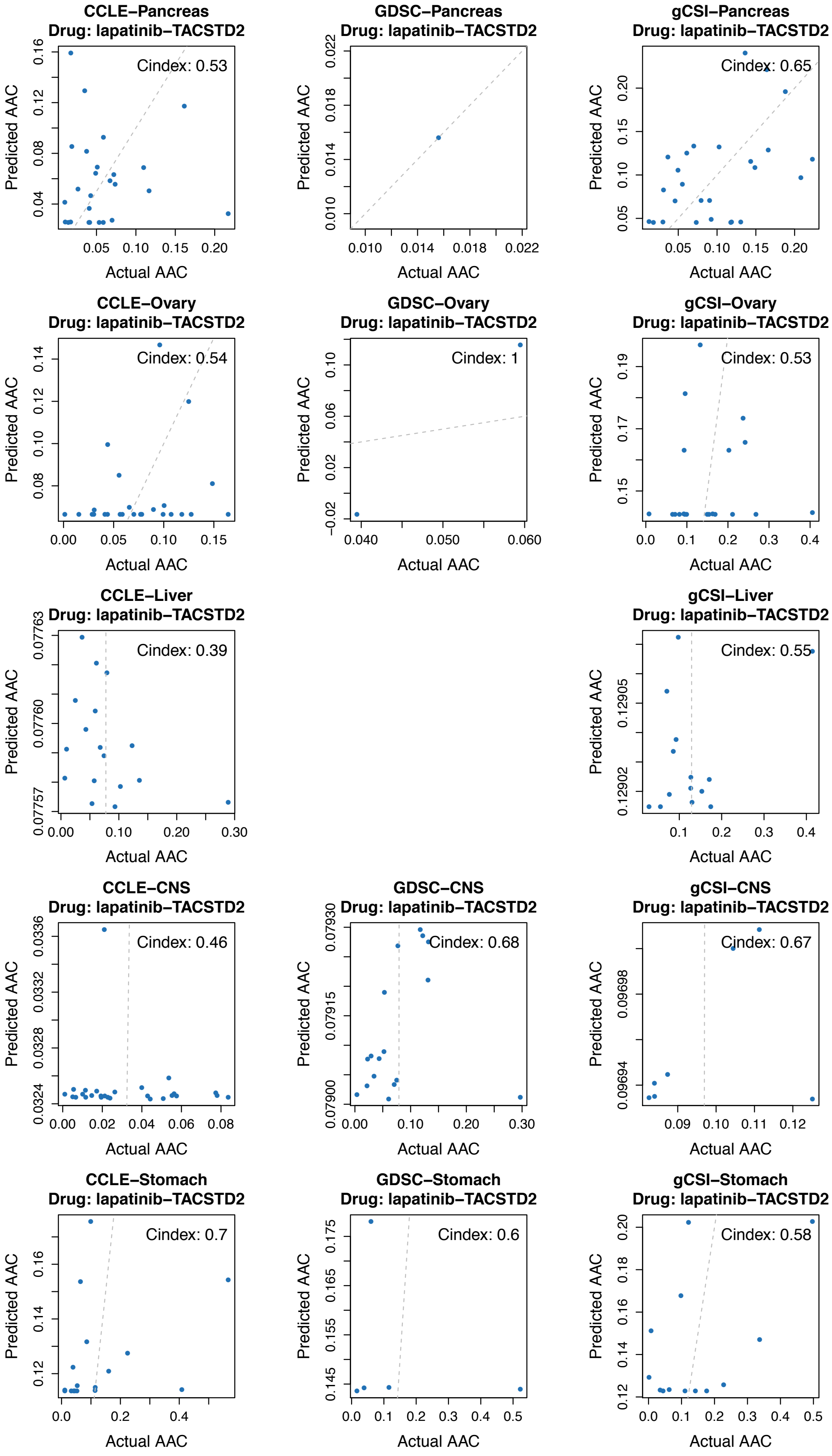

Supplementary Figure 16 (cont'd): Predicted versus actual AAC in each tissue type for the top biomarker associated with the drugs in common between gCSI, CCLE and GDSC.

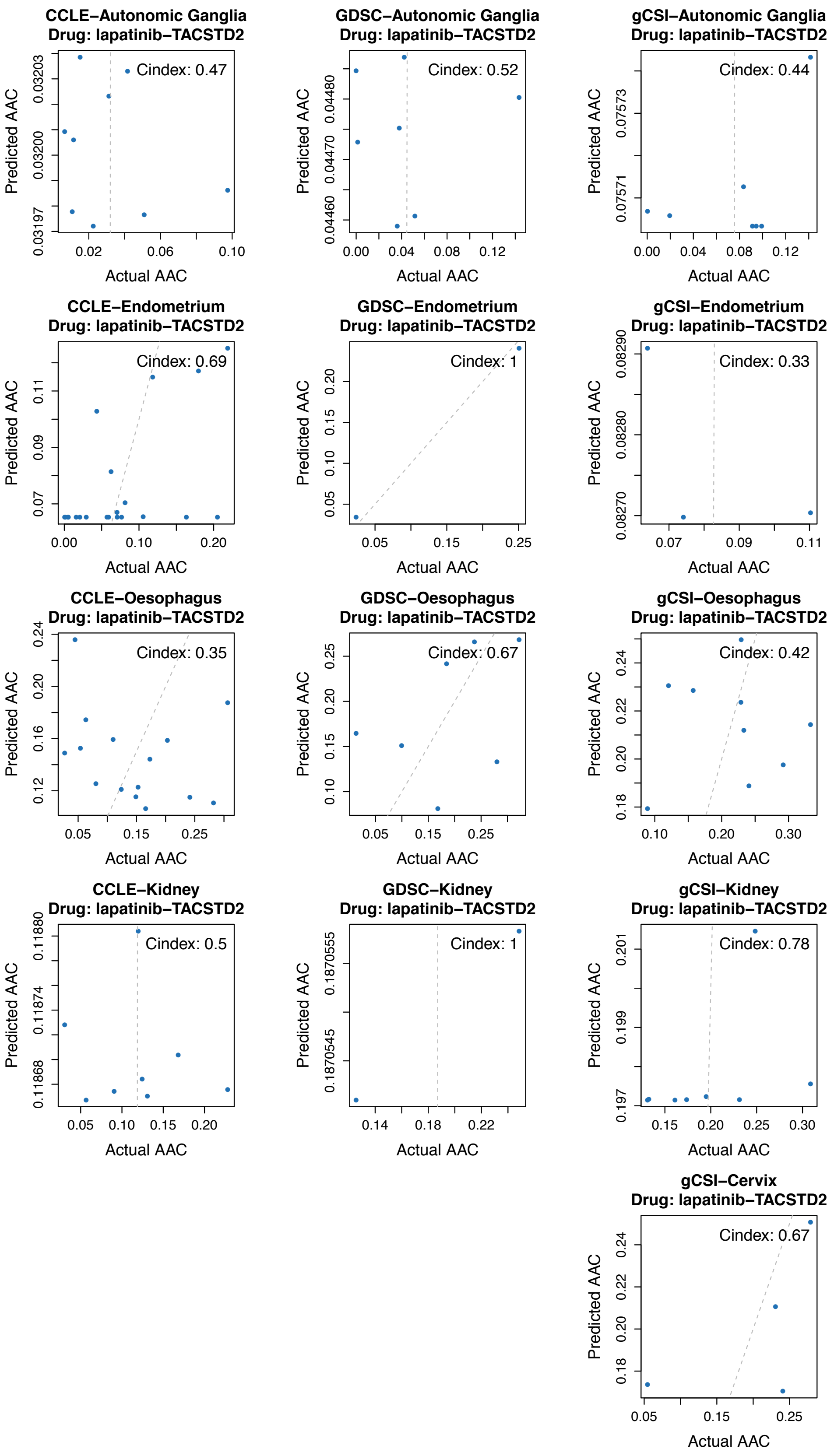

Supplementary Figure 16 (cont'd): Predicted versus actual AAC in each tissue type for the top biomarker associated with the drugs in common between gCSI, CCLE and GDSC.

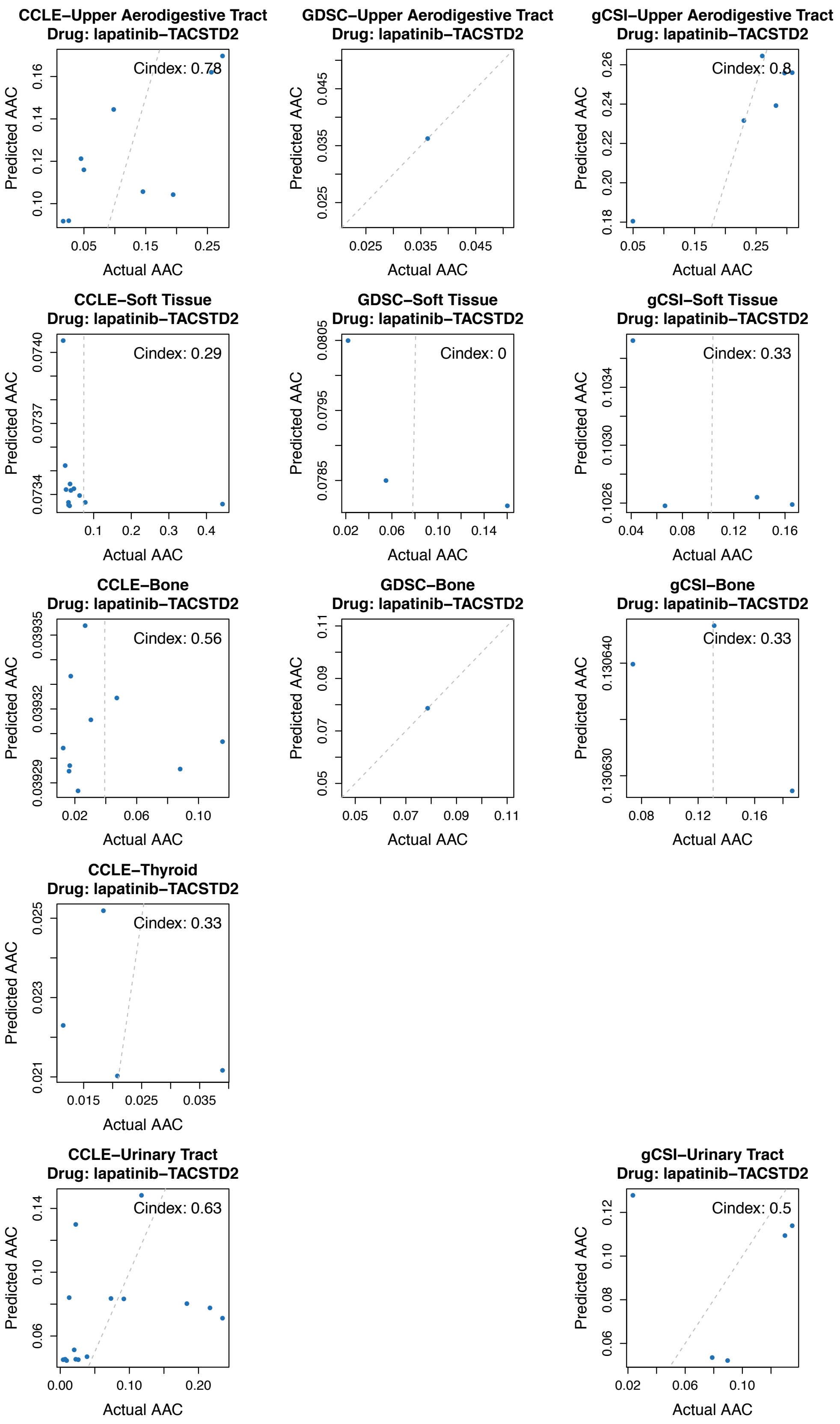

Supplementary Figure 16 (cont'd): Predicted versus actual AAC in each tissue type for the top biomarker associated with the drugs in common between gCSI, CCLE and GDSC.

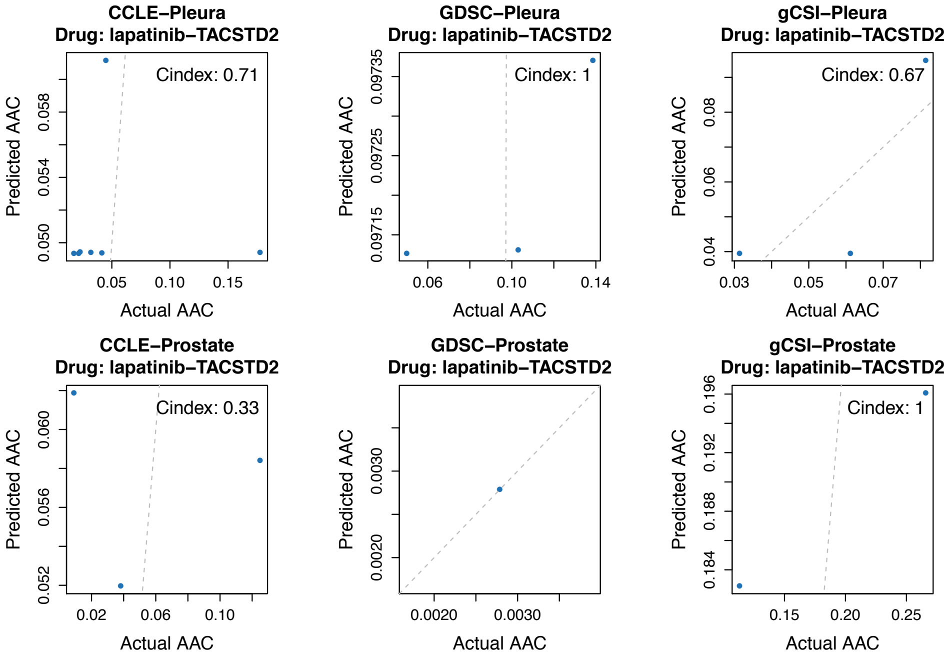

Supplementary Figure 16 (cont'd): Predicted versus actual AAC in each tissue type for the top biomarker associated with the drugs in common between gCSI, CCLE and GDSC.

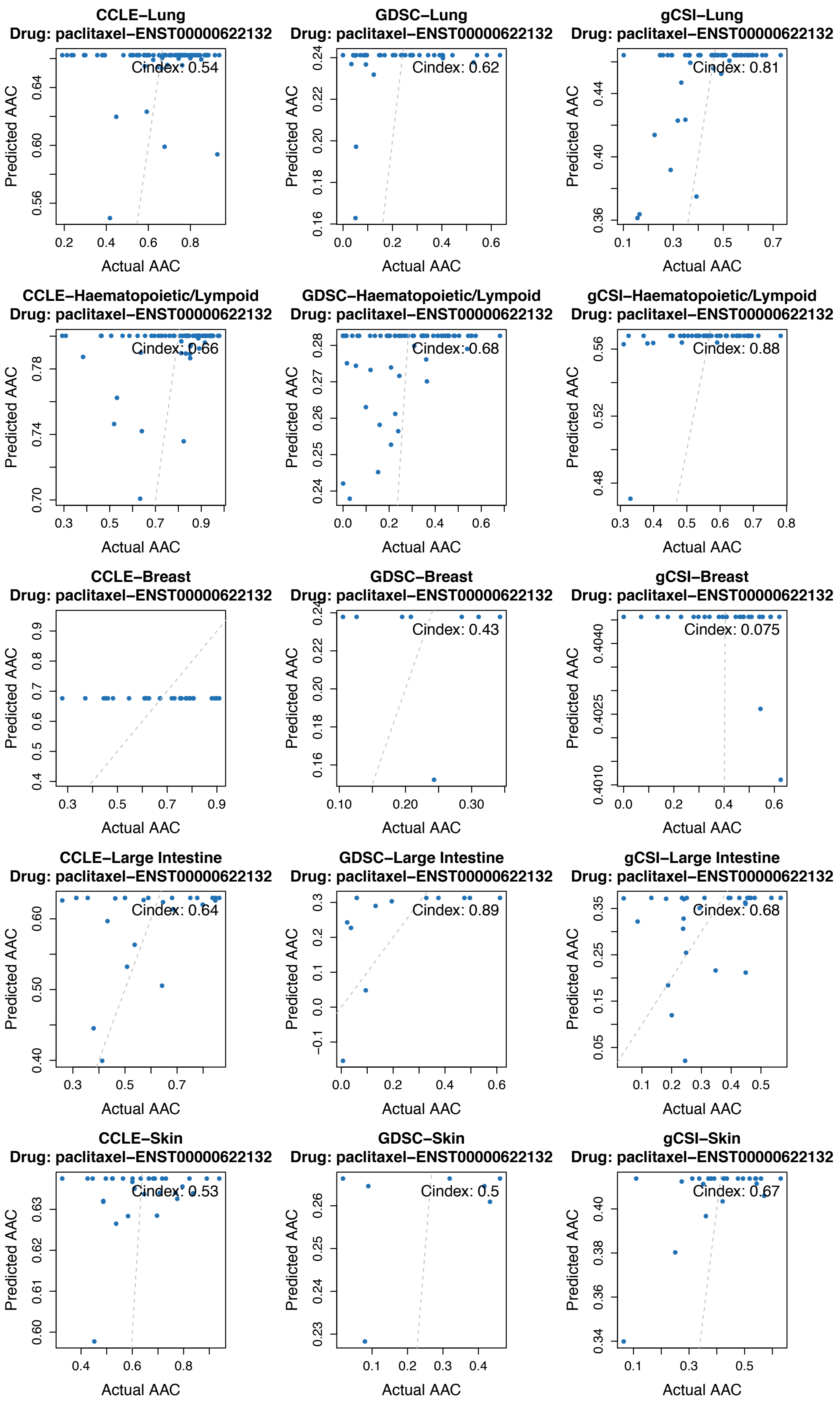

Supplementary Figure 16 (cont'd): Predicted versus actual AAC in each tissue type for the top biomarker associated with the drugs in common between gCSI, CCLE and GDSC.

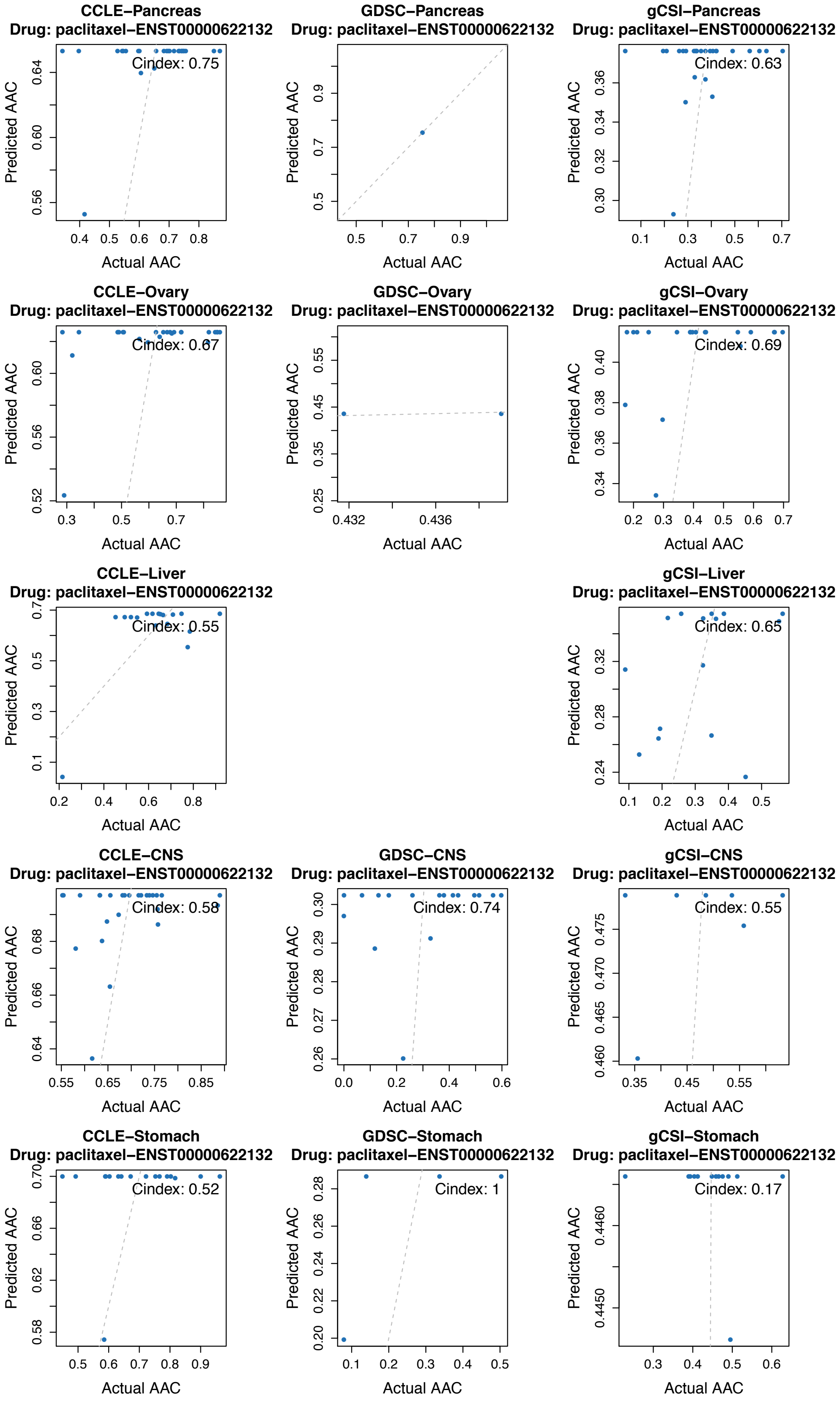

Supplementary Figure 16 (cont'd): Predicted versus actual AAC in each tissue type for the top biomarker associated with the drugs in common between gCSI, CCLE and GDSC.

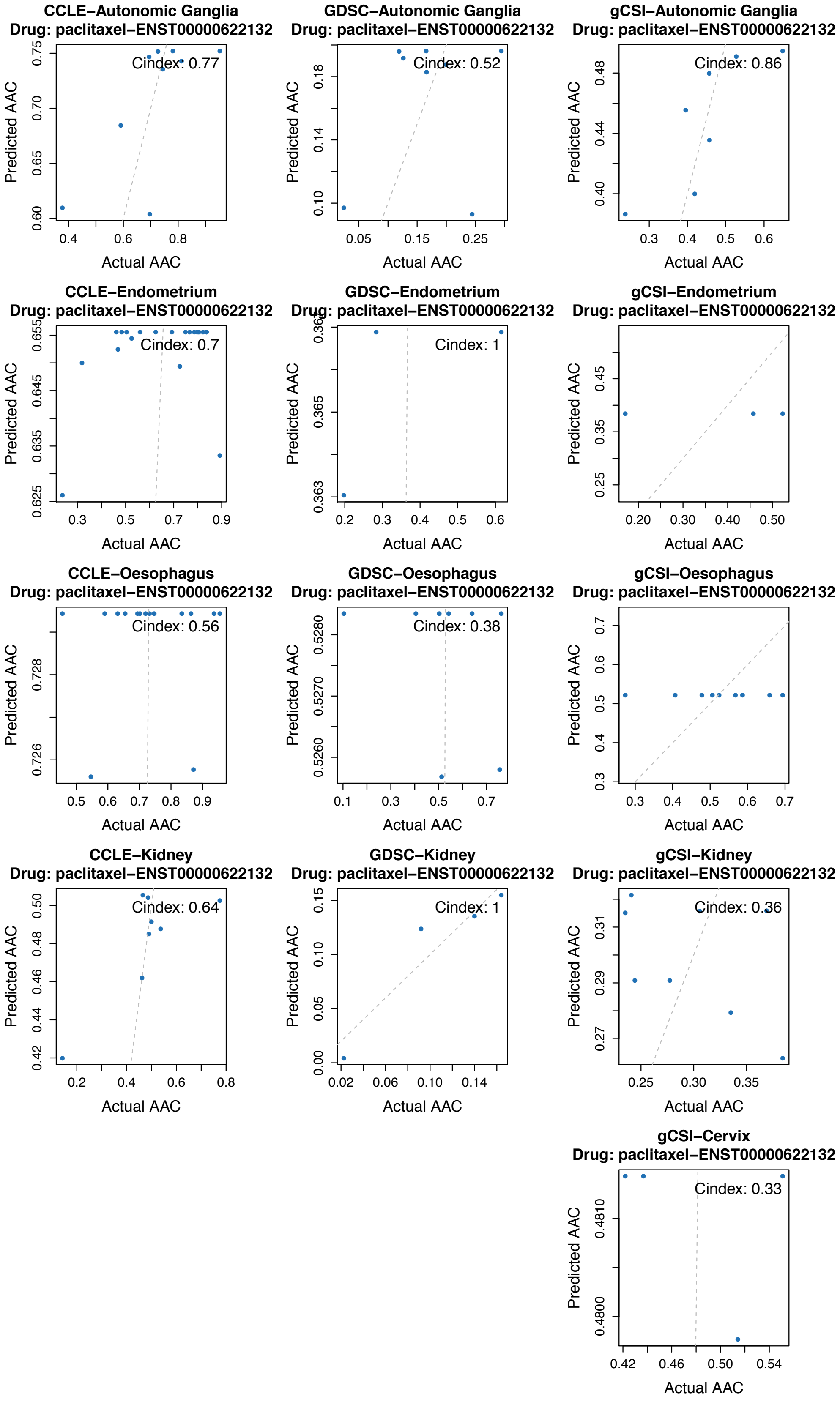

Supplementary Figure 16 (cont'd): Predicted versus actual AAC in each tissue type for the top biomarker associated with the drugs in common between gCSI, CCLE and GDSC.

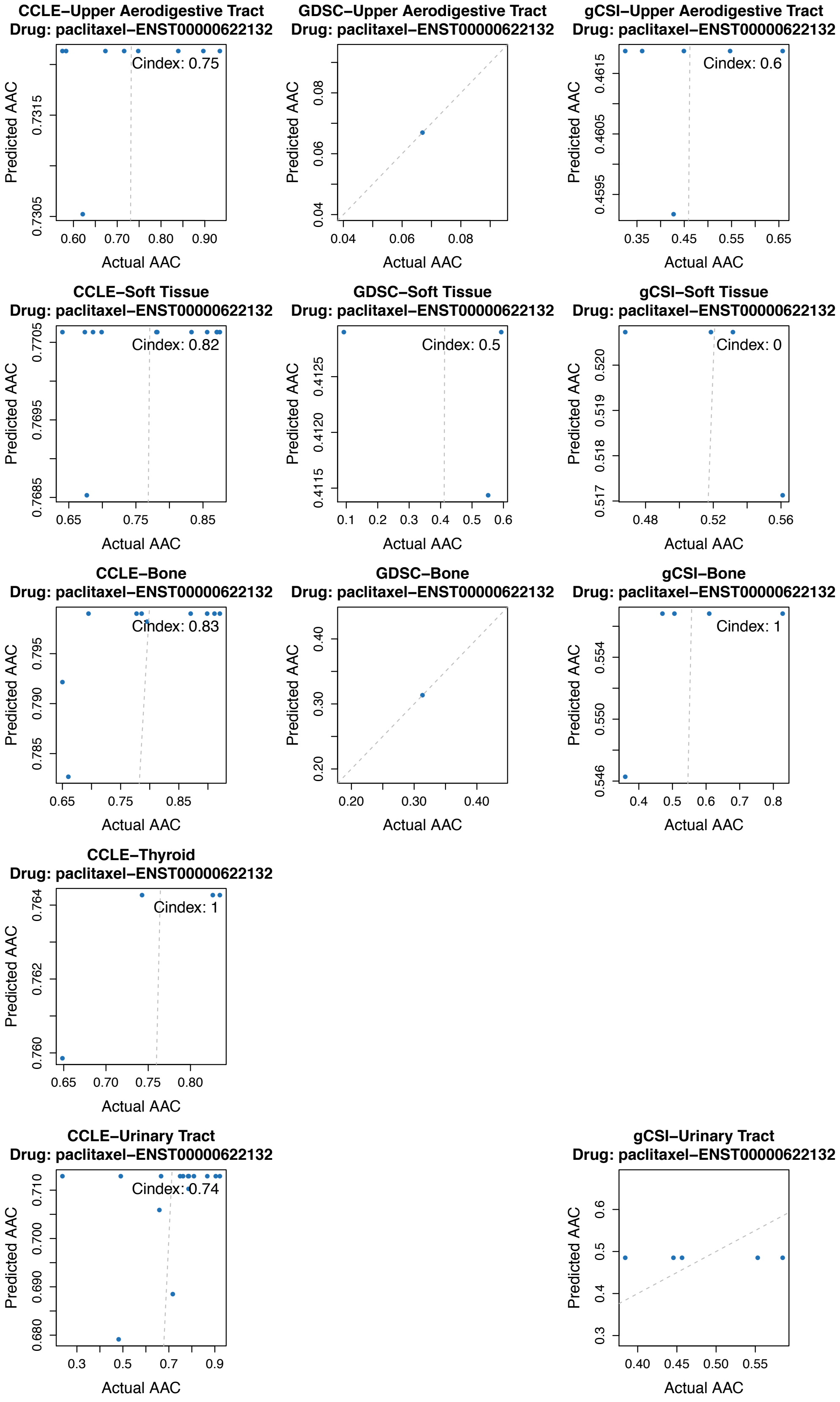

Supplementary Figure 16 (cont'd): Predicted versus actual AAC in each tissue type for the top biomarker associated with the drugs in common between gCSI, CCLE and GDSC.

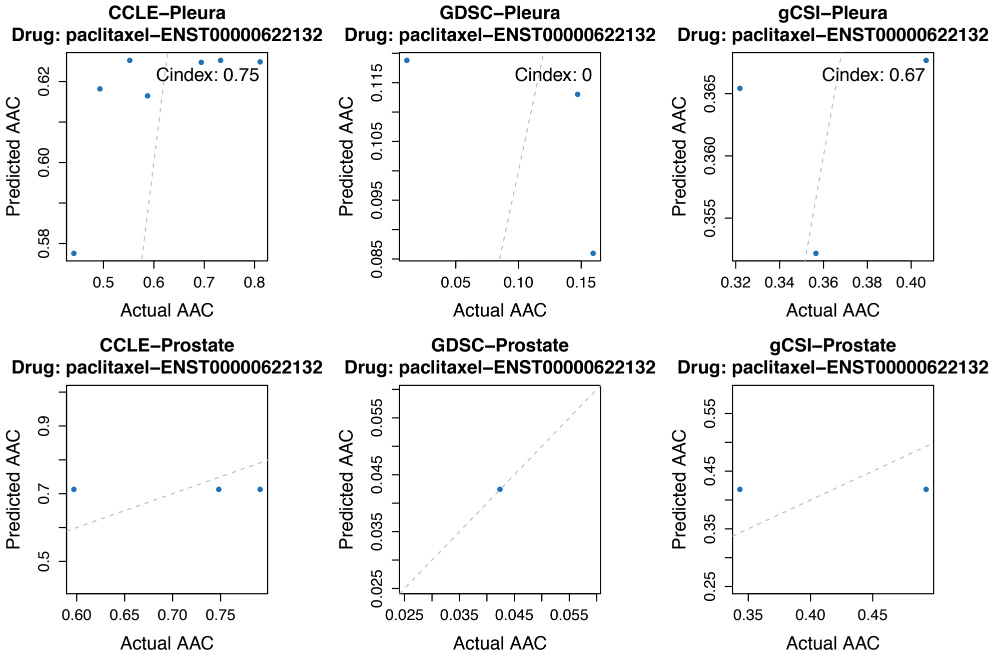

Supplementary Figure 16 (cont'd): Predicted versus actual AAC in each tissue type for the top biomarker associated with the drugs in common between gCSI, CCLE and GDSC.

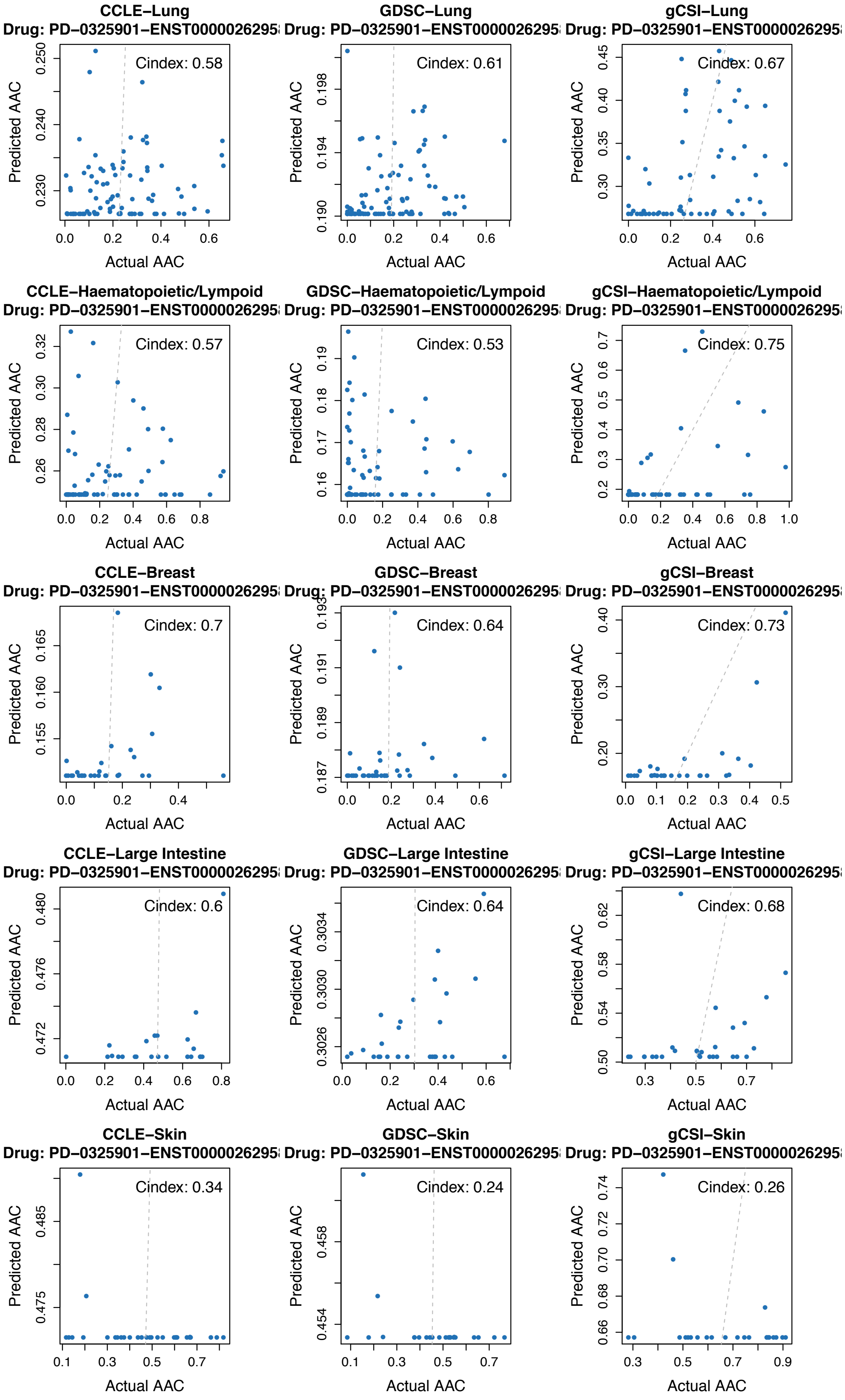

Supplementary Figure 16 (cont'd): Predicted versus actual AAC in each tissue type for the top biomarker associated with the drugs in common between gCSI, CCLE and GDSC.

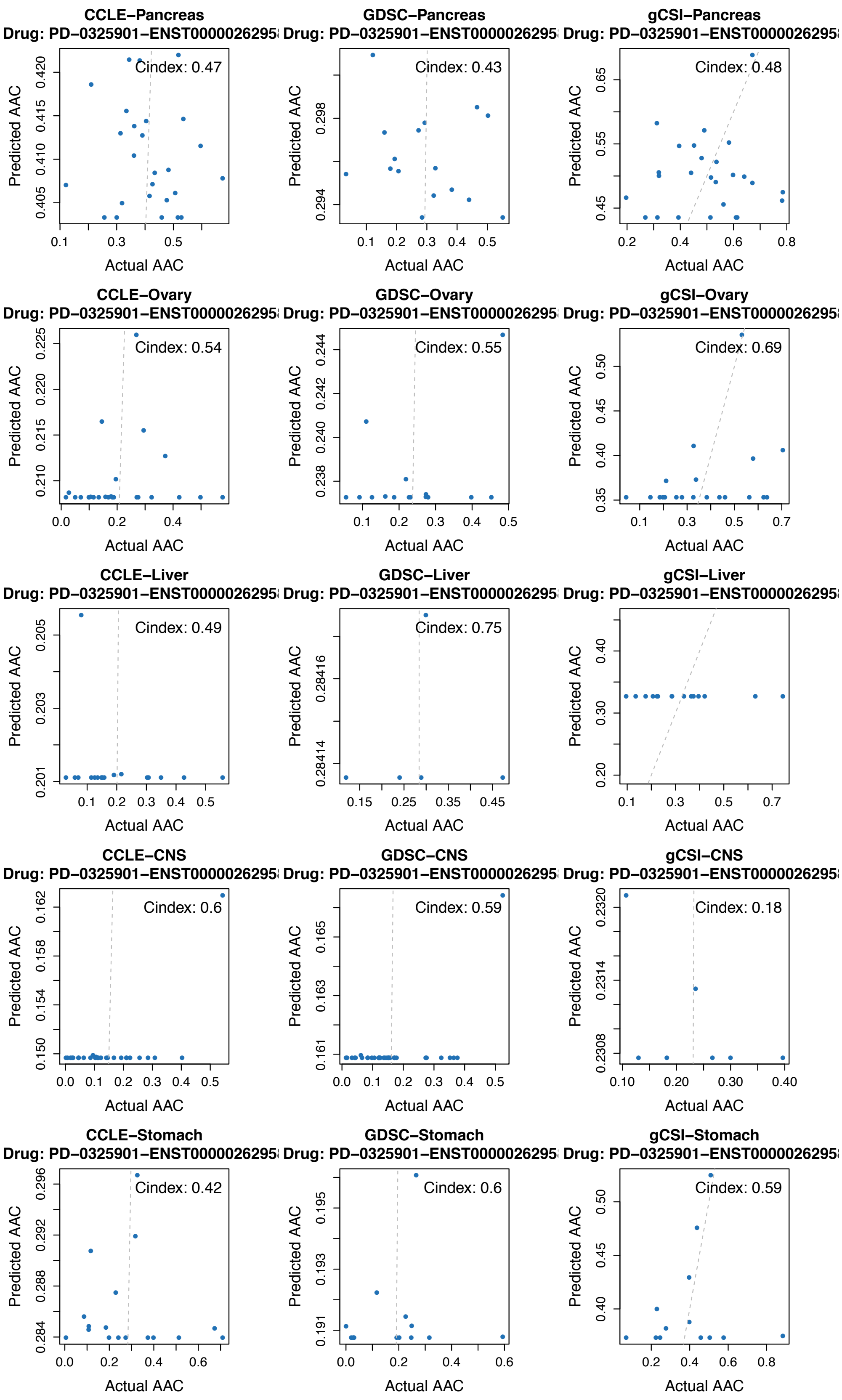

Supplementary Figure 16 (cont'd): Predicted versus actual AAC in each tissue type for the top biomarker associated with the drugs in common between gCSI, CCLE and GDSC.

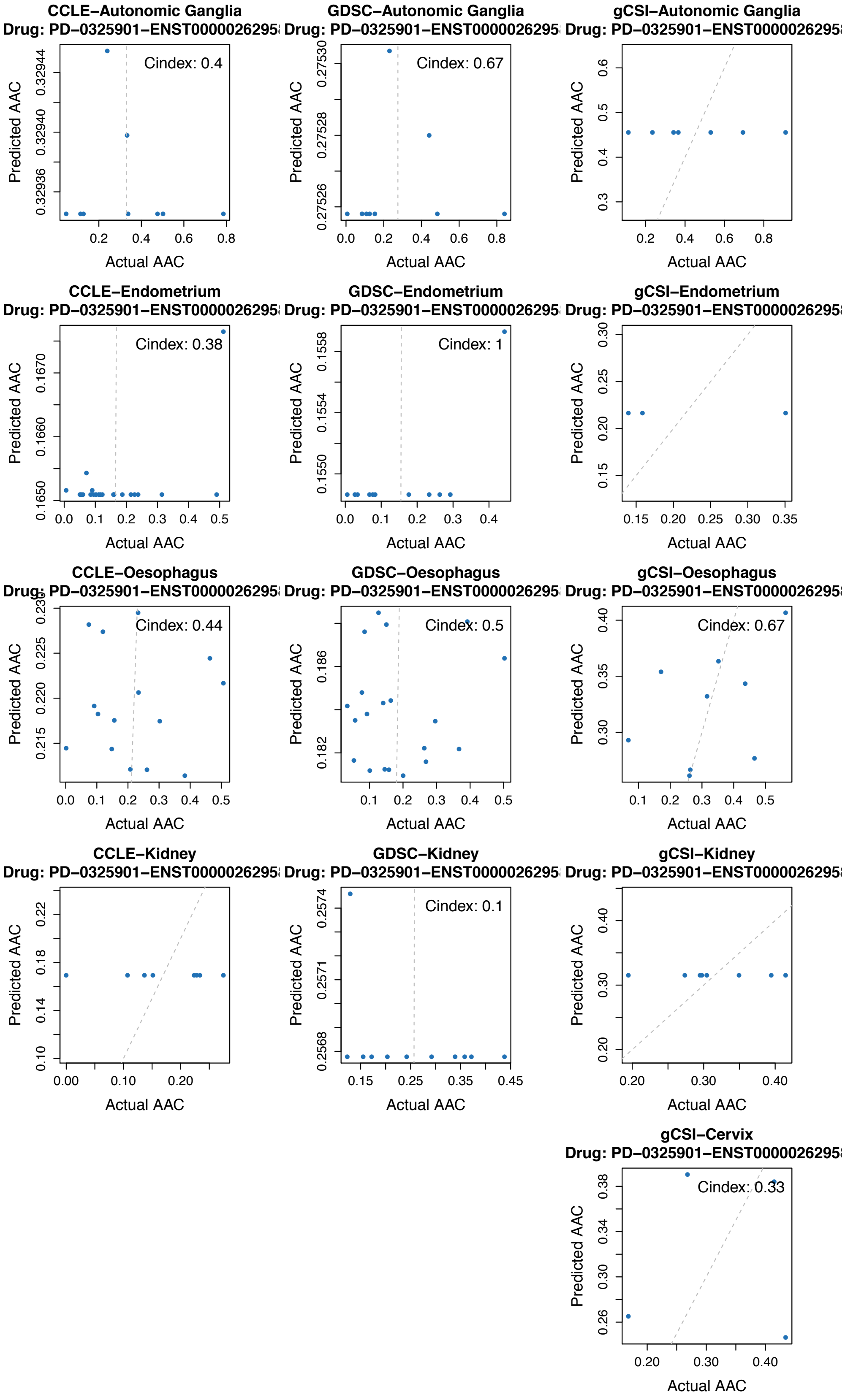

Supplementary Figure 16 (cont'd): Predicted versus actual AAC in each tissue type for the top biomarker associated with the drugs in common between gCSI, CCLE and GDSC.

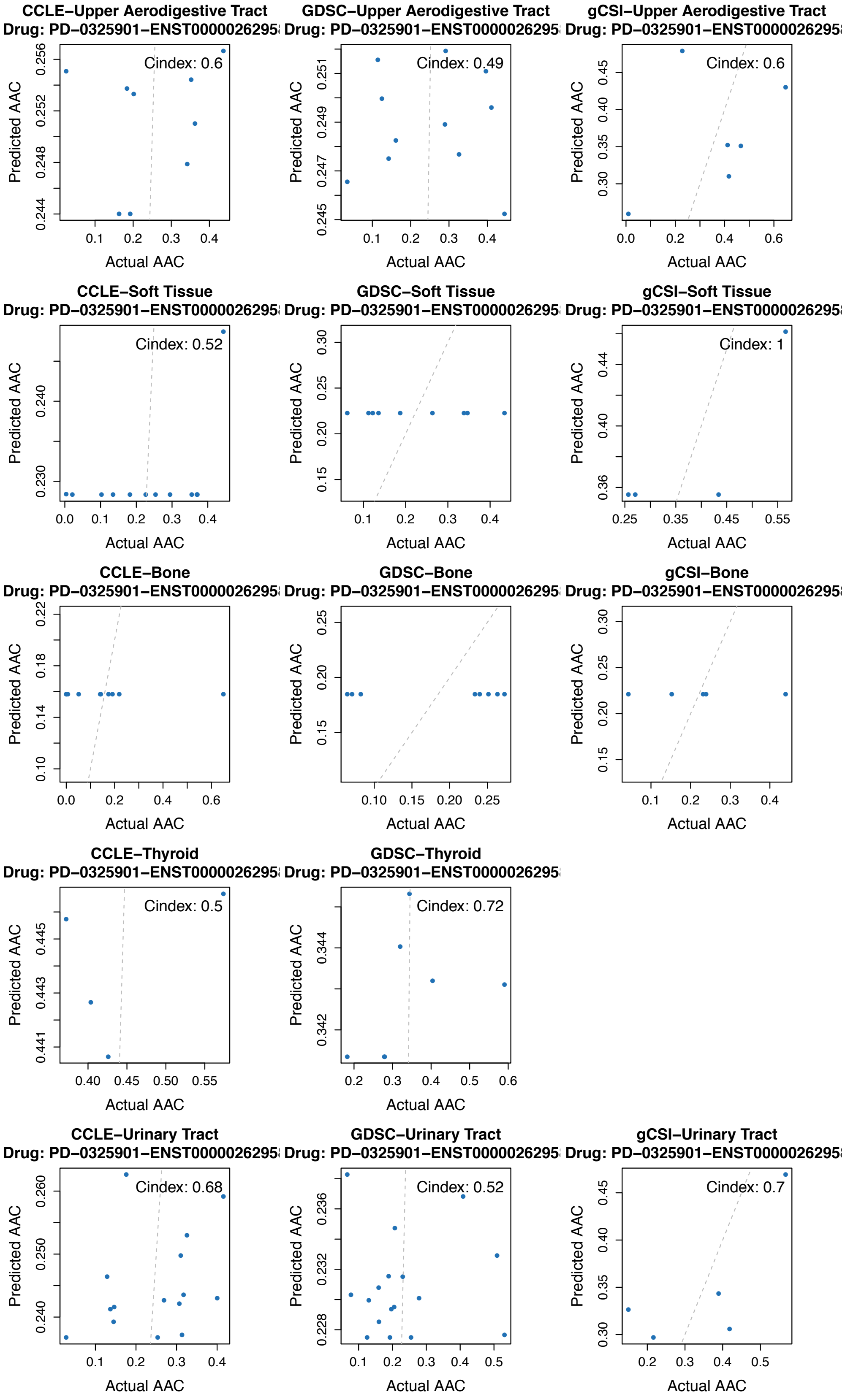

Supplementary Figure 16 (cont'd): Predicted versus actual AAC in each tissue type for the top biomarker associated with the drugs in common between gCSI, CCLE and GDSC.

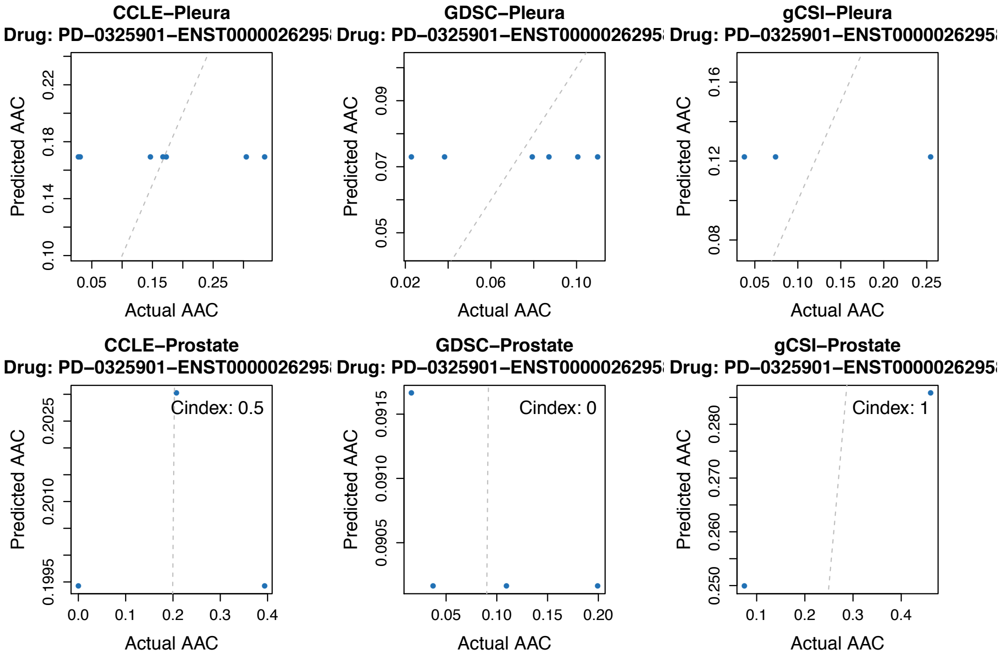

Supplementary Figure 16 (cont'd): Predicted versus actual AAC in each tissue type for the top biomarker associated with the drugs in common between gCSI, CCLE and GDSC.

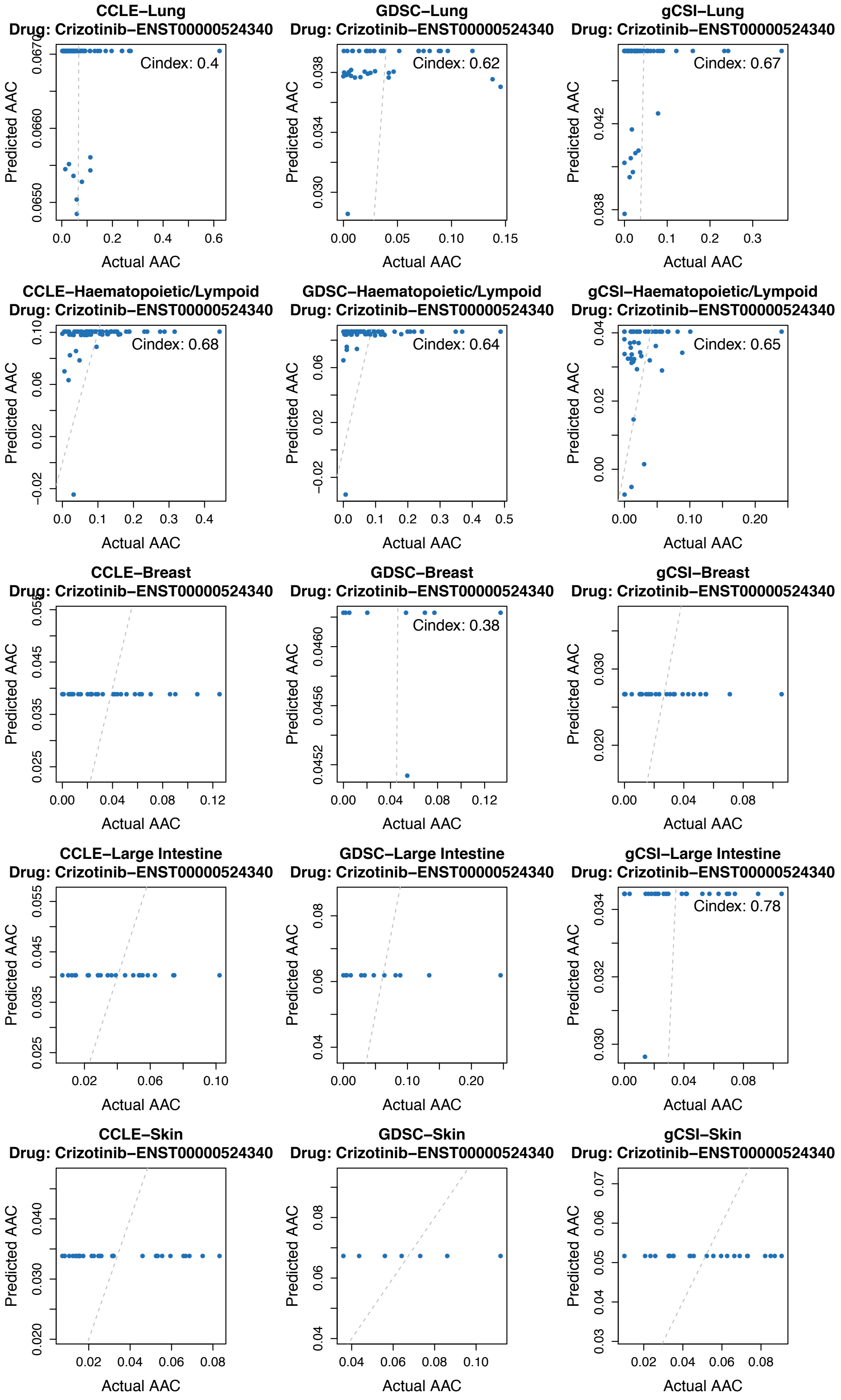

Supplementary Figure 16 (cont'd): Predicted versus actual AAC in each tissue type for the top biomarker associated with the drugs in common between gCSI, CCLE and GDSC.

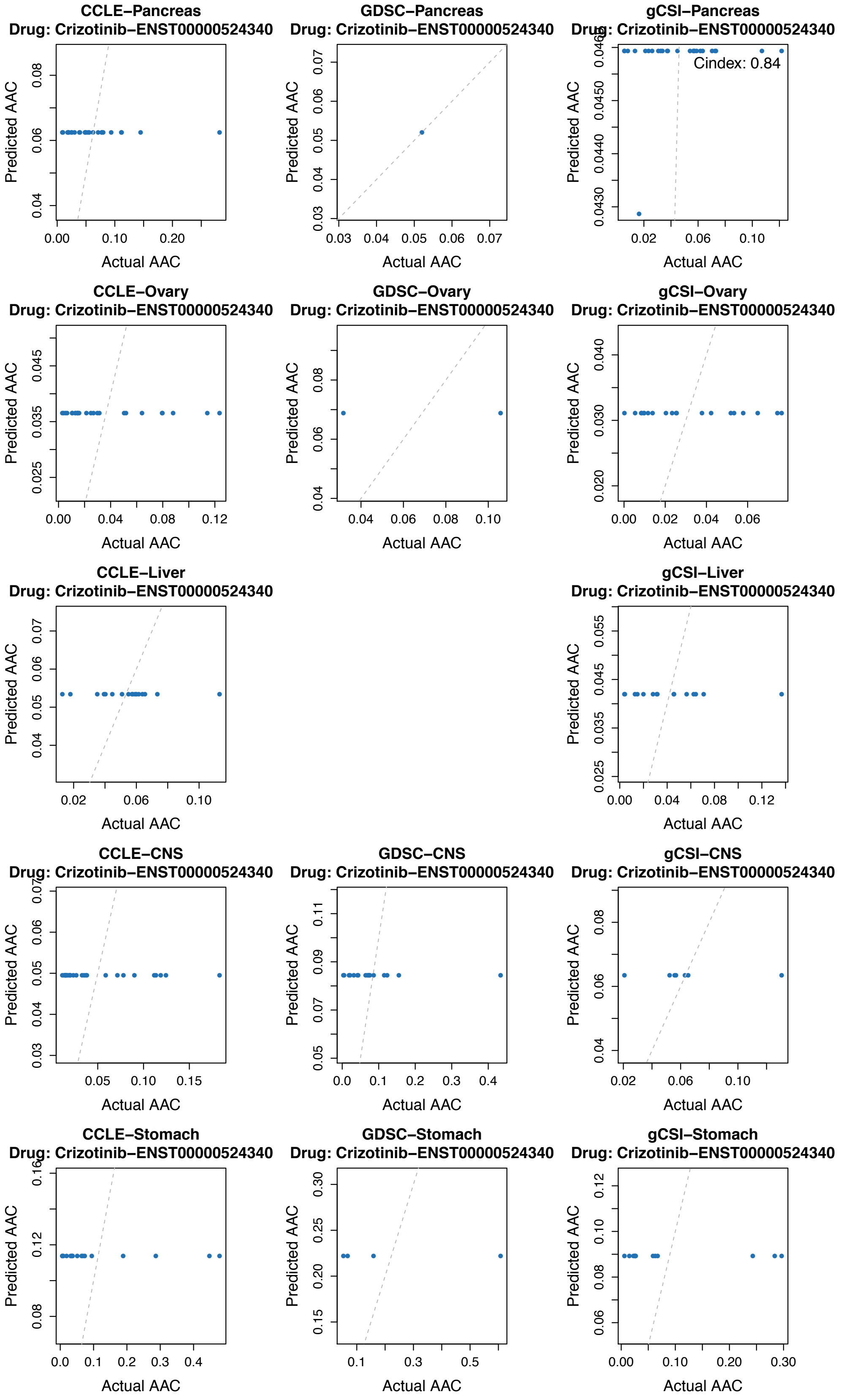

Supplementary Figure 16 (cont'd): Predicted versus actual AAC in each tissue type for the top biomarker associated with the drugs in common between gCSI, CCLE and GDSC.

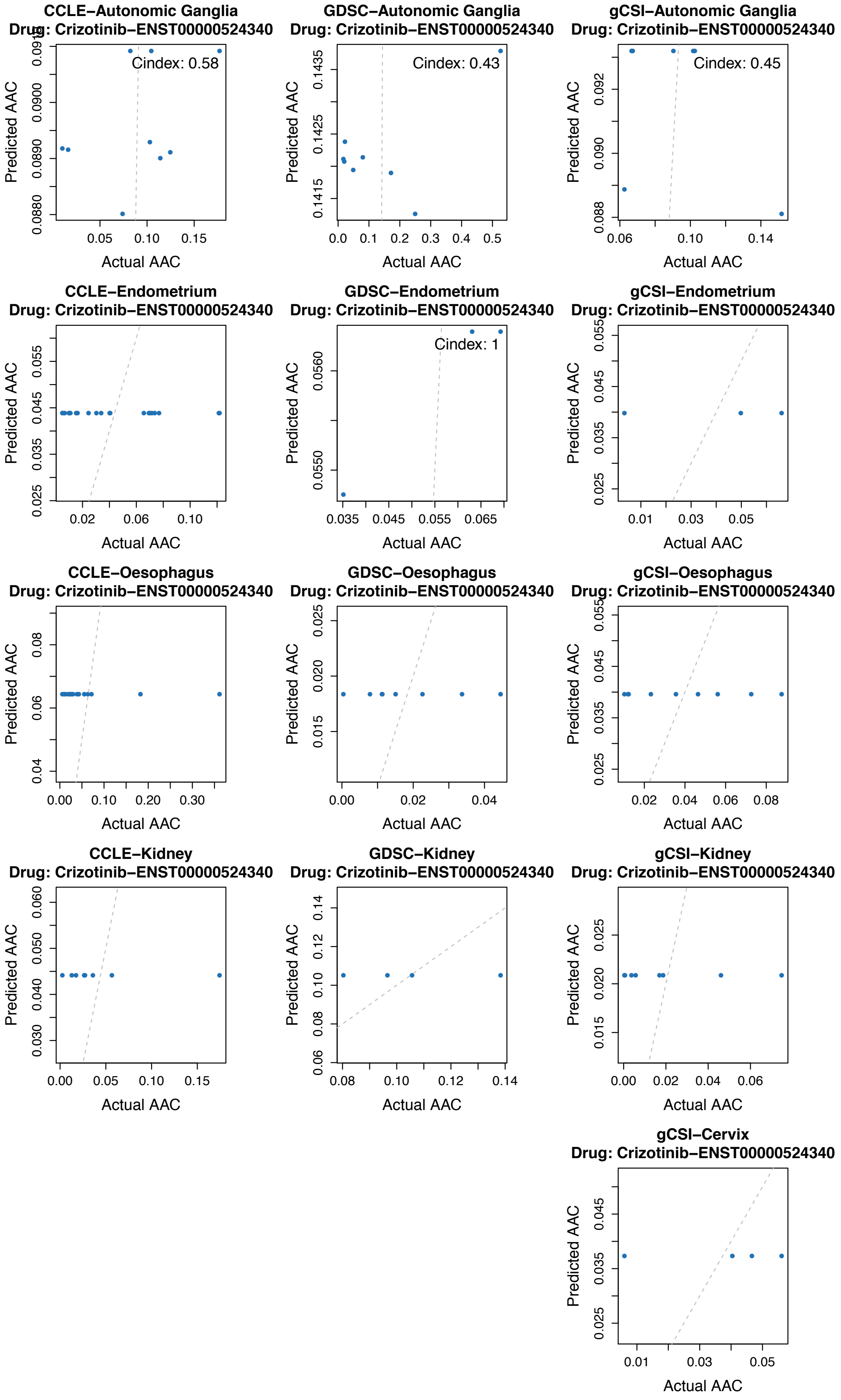

Supplementary Figure 16 (cont'd): Predicted versus actual AAC in each tissue type for the top biomarker associated with the drugs in common between gCSI, CCLE and GDSC.

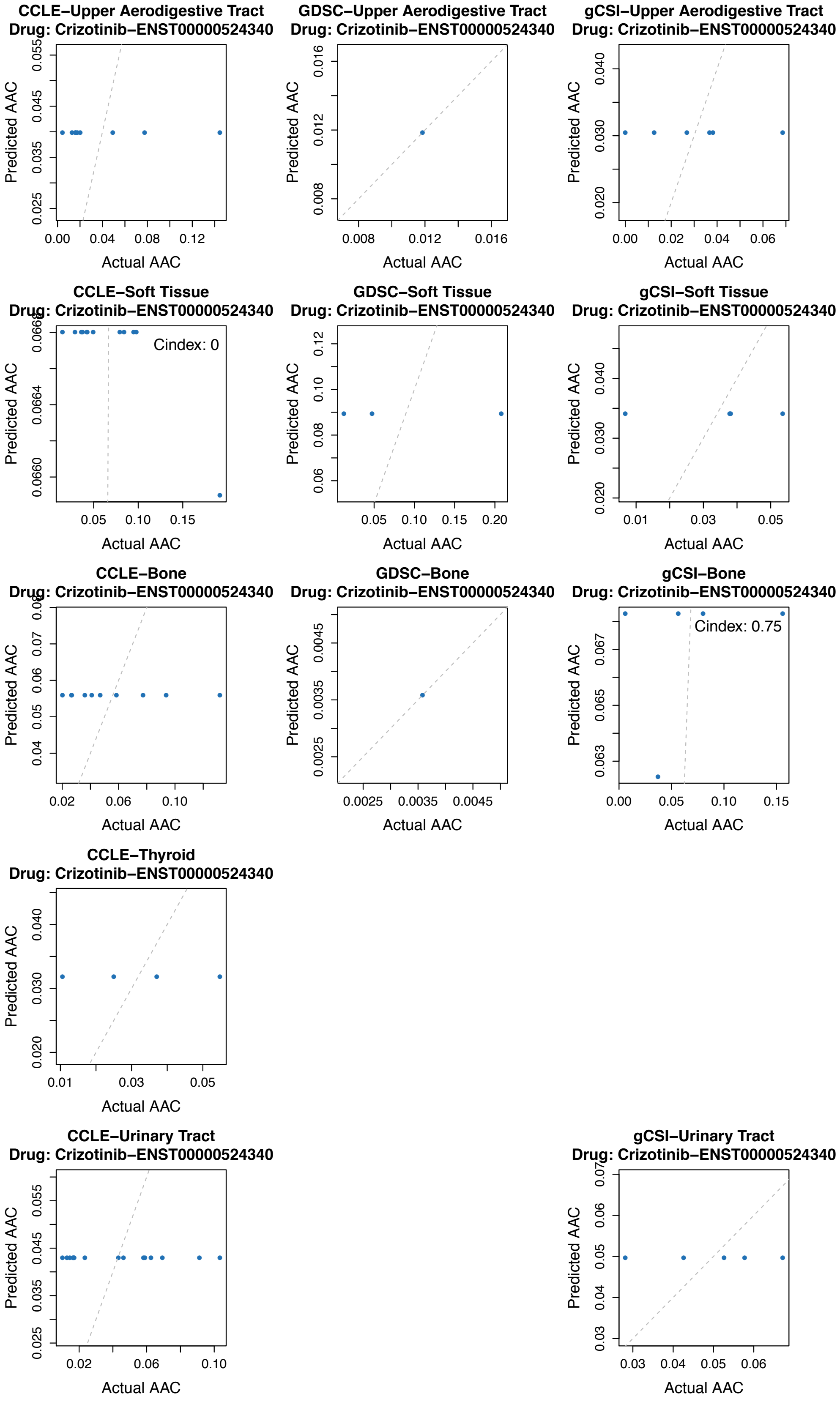

Supplementary Figure 16 (cont'd): Predicted versus actual AAC in each tissue type for the top biomarker associated with the drugs in common between gCSI, CCLE and GDSC.

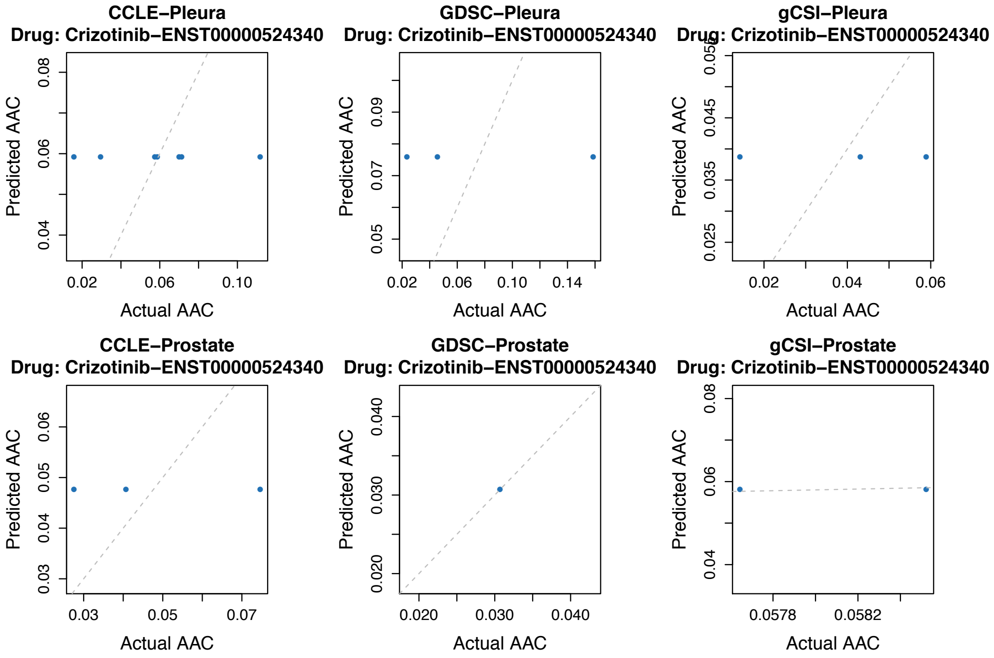

Supplementary Figure 16 (cont'd): Predicted versus actual AAC in each tissue type for the top biomarker associated with the drugs in common between gCSI, CCLE and GDSC.
