# Supplementary material for: Gene isoforms as expression-based biomarkers predictive of drug response in vitro
Source: Nat Commun. 2017 Oct 24;8:1126. doi: 10.1038/s41467-017-01153-8 (PMC5655668; doi:10.1038/s41467-017-01153-8)
Supplement: Supplementary file 3 — Description of Additional Supplementary Files [file 41467_2017_1153_MOESM3_ESM.pdf]

Description of Supplementary Files

File name: Supplementary Data 1
Description: List of significant associations identified in the training sets.

File name: Supplementary Data 2
Description: List of validated pan-cancer biomarkers in gCSI.

File name: Supplementary Data 3
Description: List of pre-validated breast biomarkers in GRAY.

File name: Supplementary Data 4
Description: List of validated breast biomarkers in UHN.

File name: Supplementary Data 5
Description: List of drugs in GDSC, CCLE, gCSI, GRAY and UHN.

File name: Supplementary Data 6
Description: List of cell lines screened in GDSC, CCLE, gCSI, GRAY and UHN.
